# Supplementary material for: Identifying and prioritising unanswered research questions for people with hyperacusis: James Lind Alliance Hyperacusis Priority Setting Partnership
Source: BMJ Open. 2019 Nov 21;9(11):e032178. doi: 10.1136/bmjopen-2019-032178 (PMC6886978; doi:10.1136/bmjopen-2019-032178)
Supplement: Supplementary data [file bmjopen-2019-032178supp001.pdf]

SUPPLEMENTAL MATERIALS

Supplemental appendix 1. Steering Group of the Hyperacusis Priority Setting Partnership.

Steering group member	Role
**James Lind Alliance support and guidance**	
Toto Anne Gronlund	Independent chair, representing James Lind Alliance
Katherine Cowan	Final workshop Facilitator, The James Lind Alliance
Sheela Upadhyaya	Final workshop Facilitator, The James Lind Alliance
**Non-voting members**	
Kathryn Fackrell	Study coordinator and Information specialist, Researcher, University of Nottingham
Derek Hoare	Researcher, University of Nottingham
Helen Henshaw	Researcher, University of Nottingham
**Patient representatives**	
Linda Stratmann	User organisation, founder and co-moderator of Facebook group Hyperacusis Support & Research, and person with lived experience
Tracey Pollard	Action on Hearing Loss patient representative
Nic Wray	Person with lived experience
Carolyn Farrell	Person with lived experience
Mike Meadows	Person with lived experience
Hilary Hodgson	Parent
Sarah Chapman	Cochrane UK Knowledge Broker
**Clinical representatives**	
Carol MacDonald	CBT psychotherapist and Clinical Psychology Lecturer
David Baguley	Clinical Scientist (audiology)
Jacqueline Sheldrake	Audiologist
John Phillips	ENT surgeon
Josephine Marriage	Paediatric audiologist
Peter Byrom	Audiologist
Rosie Kentish	Clinical Psychologist
Veronica Kennedy	Audiovestibular Physician

Supplemental appendix 2: Partner organisations

Charity organisations

Action on Hearing Loss

Space 4 Autism

Professional organisations

British Society of Audiology
British Association of Audiovestibular Physicians
National Institute of Health Research Nottingham Biomedical Research Centre

Social media outlets
Facebook group Hyperacusis Support & Research
National Institute of Health Research Nottingham Biomedical Research Centre Facebook
Study-dedicated twitter account (@HyperacusisJLA)

Supplemental appendix 3: Interim prioritisation survey

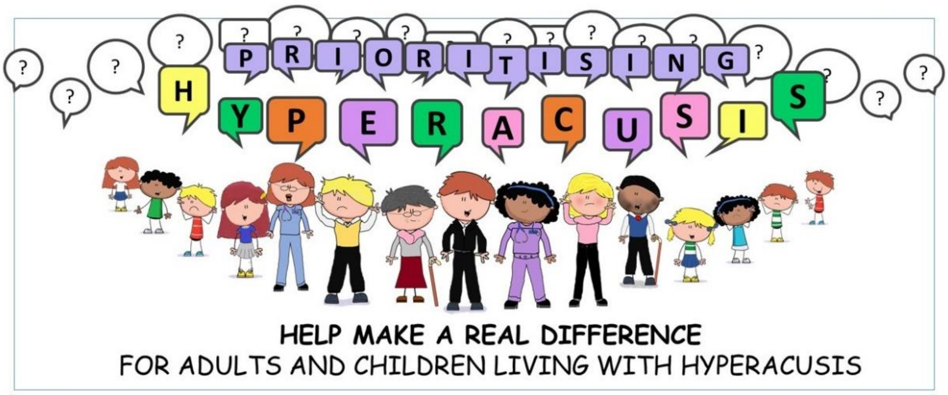

Welcome to this Hyperacusis James Lind Alliance Priority Setting Partnership survey.

Thank you for agreeing to participate!

What is hyperacusis?

Hyperacusis is a hearing disorder involving an increased sensitivity or decreased tolerance to everyday sound at levels that would not trouble most individuals. For the person experiencing hyperacusis everyday sounds can be unpleasant, intense, frightening, painful, and overwhelming and can cause anxiety affecting their quality of life.

We need you to vote for your Top 10 research questions about hyperacusis

We want to identify the Top 10 questions that research should target. Our first survey asked for people with lived experience of hyperacusis, parents, carers, teachers, members of the public and healthcare professionals who work with and support those who experience hyperacusis to submit any questions they had about hyperacusis.

In this survey you have the opportunity to choose 10 "unanswered" questions about hyperacusis that you think are the most important for research to target. Anyone with experience of hyperacusis (except researchers) can complete this survey. You do not have to have taken part in any previous studies.

The survey should not take longer than 15 minutes to complete.

Why do this?

Your responses will help us and other organisations to target research and funding for hyperacusis. This really is a unique opportunity to have your say and make sure that research addresses questions that matter to you and others like you.

Find out how on the next page...

Continue

Thank you for agreeing to complete this survey. Before we begin…

We just need to know your experience with hyperacusis to make sure that we understand the voting patterns by a wide range of people with different experiences. Anyone with experience of hyperacusis can complete this survey!

Which of the following best describes you?

Please select all the groups that apply to you. For example, if you are a healthcare professional and a parent of a child with hyperacusis then select both.

- ☐ Person with hyperacusis currently or in past
- ☐ Carer of someone with hyperacusis
- ☐ Parent of a child with hyperacusis
- ☐ Family or friend of someone with hyperacusis
- ☐ Educational professional, e.g. school teacher, lecturer
- ☐ Healthcare professional
- ☐ Other

Continue

Instructions

On the left of the screen in a list of "unanswered" research questions about hyperacusis.

On the right of screen are two category boxes ("questions of interest" and "Top 10"). The questions on the left of the screen are designed for you to move them (drag and drop them using mouse) into the boxes on the right of the screen. The category boxes will expand as questions are moved into it (example shown below).

Please read through the questions carefully. We would like you to move the 10 questions you think are most important for research to target into the "Top 10" box on the right.

As you read through the questions, please move any questions you think might be important and would like to go back over into the "questions of interest" box on the right.

You can swap and change questions from all of the boxes as often as you like. There is no right or wrong answer, just chose the questions that you think are most important for research to answer.

We are not asking you to rank the questions so you can place them in any order you like in the Top 10 group.

PLEASE make sure that 10 questions are placed in the “Top 10" box on the right. If you think there are additional questions that are important then leave them in the "questions of interest" box.

When you have selected your Top 10 questions, please click **"Finished"** in the top right corner for the next part of the survey.

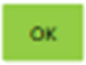

Questions

Are there different meaningful types of hyperacusis?
Does diet have an effect on hyperacusis?
Does exercise/physical activity have an effect on hyperacusis?
Which hearing aid settings (for managing hearing loss) are safest for people with hyperacusis?
Are online treatments effective for hyperacusis?
Are online informational resources effective for informing people about hyperacusis?
Which interventions in a school setting are useful for children with hyperacusis?
What are the most effective and acceptable strategies to raise awareness about hyperacusis?
Is private healthcare for hyperacusis more effective than NHS usual care?
Which management approach (e.g. assessment and treatment) for hyperacusis is most effective for people with learning disabilities?
What are the precautions that a dentist should use for their patients who have hyperacusis?
Which hyperacusis self-management techniques help to cope with invasive procedures (e.g. having dental work)?
Which health professionals should work together to provide the best care for adults with hyperacusis?
Is attention deficit hyperactivity disorder associated with hyperacusis?
Which strategies would make services more accessible?
What is the most effective treatment approach for hyperacusis in adults?
How should hyperacusis be assessed in different populations?
Which assessments should be conducted in the workplace to monitor and adjust for staff who have or may have hyperacusis?
What are the indicators for specialist care (e.g. psychological therapy) for someone with hyperacusis?
What is the most effective treatment approach for hyperacusis in children?
What are the early warning signs of hyperacusis?
What care is most effective for recent onset/acute hyperacusis?
Which treatment approaches are most effective for different types or severities of hyperacusis?
What is the association between hyperacusis and dementia?
Does nerve damage cause the pain associated with hyperacusis?
Is the success of hyperacusis treatment affected by the level of support provided by caregivers (e.g. parents, spouses, partners, family)?
Would restoring hearing (e.g. regenerating nerve cells) improve hyperacusis?
What is the best way of using sound in therapy for hyperacusis?
Does learned behaviour from family members with hyperacusis increase the risk of hyperacusis developing?

Which drugs are effective for hyperacusis?
What educational information and support is effective for hyperacusis?
Is Tinnitus Retraining Therapy effective for hyperacusis?
Is Neuromonics™ effective for hyperacusis?
Are any surgical interventions effective for hyperacusis?
What is the prevalence of hyperacusis in a general population and other specific populations (e.g. people with autism, mental health issues, learning disabilities, hearing loss)?
Is pain hyperacusis more related to hearing loss than other types of hyperacusis?
Can/should there be monitoring or screening for hyperacusis?
Can early diagnosis reduce the risk of hyperacusis getting worse?
What is the natural history (onset and changes over time) of hyperacusis?
Are hearing aids (for managing hearing loss) an effective treatment for hyperacusis?
Is there an association between hearing loss and hyperacusis?
Is peer support (e.g. support groups, mentors, families) effective for management of hyperacusis?
Does avoidance of sound improve hyperacusis or make it worse?
Is stress management effective for hyperacusis?
Is hyperacusis a symptom, a condition or a disability?
What is the level of public/employer awareness of hyperacusis?
What should be the maximum volume (e.g. in movies, concerts, headphones) to accommodate people with hyperacusis?
Can noise exposure cause hyperacusis (or make it worse)?
What preventive measures (e.g. educational information, warning labels) reduce the risk of hyperacusis developing or getting worse?
Are there certain personality traits in those prone to hyperacusis?
Which management approach (e.g. assessment and treatment) is most effective for people with hyperacusis and dementia?
What are the risk factors for developing hyperacusis or making it worse?
Is there a link between hyperacusis and non-ear-related conditions (e.g. Fibromyalgia, multiple sclerosis, diabetes, chronic fatigue, pernicious anaemia)?
Which health professionals should work together to provide the best care for children with hyperacusis?
What is the essential knowledge/training required for health professionals to appropriately refer or effectively manage hyperacusis?
Which psychological therapy (e.g. counselling, Cognitive Behavioural Therapy, mindfulness) is most effective for hyperacusis?
Is hyperacusis associated with middle ear pathology?
What is the relationship between hyperacusis and autism?
What is the relationship between mental health and hyperacusis?

Is hyperacusis linked to other sensitivities/conditions?
Is there an association between hyperacusis and other ear-related conditions (e.g. superior canal dehiscence syndrome, Meniere's, Waardenburg syndrome, vertigo, vestibular migraines)?
Is hyperacusis linked with trauma (e.g. brain or neck trauma, a traumatic event)?
Does stress cause or make hyperacusis worse?
Why does hyperacusis fluctuate?
What is the role of fear in hyperacusis?
Which drugs cause hyperacusis?
Is there a genetic element to hyperacusis?
What is the best work/study/healthcare environment to support people with hyperacusis?
Are any complementary (relaxation, yoga, meditation, hypnotherapy) or alternative therapies (laser therapy, vibration therapy) effective for hyperacusis?
Is hyperacusis related to physical changes in the ear or brain?
Is hyperacusis due to physical or psychological issues or is it a combination of both?
What is the best information and guidance for parents dealing with hyperacusis in their children?
What area(s) of the brain and patterns of activity is/are associated with hyperacusis?
Can otitis media with effusion cause hyperacusis?
Can infections (e.g. measles, yeast, viral) cause hyperacusis?
Can ear care (suctioning, syringing, swabbing) cause hyperacusis?
Do dental problems cause hyperacusis?
What is the best way to differentiate hyperacusis from other hearing conditions (e.g. recruitment, misophonia, Meniere's, tinnitus)?
Can hyperacusis be caused or made worse (e.g. by hormonal changes) in pregnancy?
Do musculoskeletal problems cause hyperacusis?
What are the 'red flags' for serious underlying conditions in hyperacusis?
Which criteria should be met to diagnose hyperacusis in adults/children?
Do any drugs prevent hyperacusis?
Which self-help interventions are effective for hyperacusis?
What management approach for hyperacusis is most effective for adults/children with autism?

Just about finished…a little bit more about you…

It is important we find out about a little about you, to make sure that we have a wide range of people with different experiences complete the survey.

What is your age or the age of your child with hyperacusis?

- ☐ under 10
- ☐ 10 - 20
- ☐ 21 - 30
- ☐ 31 - 40
- ☐ 41 - 50
- ☐ 51 - 60
- ☐ 61 - 70
- ☐ 71 - 80
- ☐ 80+

How would you describe your gender?

- ☐ Male
- ☐ Female
- ☐ I would describe it in another way

Healthcare professional only: What is your main profession?

For example, GP, ENT, Audiologist, clinical psychologist.

- ☐ Audiologist
- ☐ Audiovestibular Physician
- ☐ Chiroptactor
- ☐ Clinical Psychologist
- ☐ Clinical scientist
- ☐ ENT
- ☐ Hearing therapist
- ☐ Hynotherapist
- ☐ Paediatric audiologist
- ☐ Paediatrician
- ☐ Psychiatrist
- ☐ Nutritionist
- ☐ Other______________________________

Thank you for completing this survey

Would you like to help us in the next stage of this study?

The final stage of project will be a face-to-face consensus meeting with 30 people to discuss the results from this survey and agree the Top 10 research questions. We are delighted to announce that this meeting will be held in the picturesque surroundings of the University of Nottingham (UK) on Monday 23rd July (10.00 - 17.00). **We need people with lived experience of hyperacusis, parents, carers, teachers, members of the public and healthcare professionals with experience of hyperacusis to participate in this meeting.** If you live in the UK and would like to participate, please contact us here to register for the meeting: JLAhyperacusis@nottingham.ac.uk. Places are available on a first come, first served basis. Travel for the meeting will be reimbursed and accommodation will be provided.

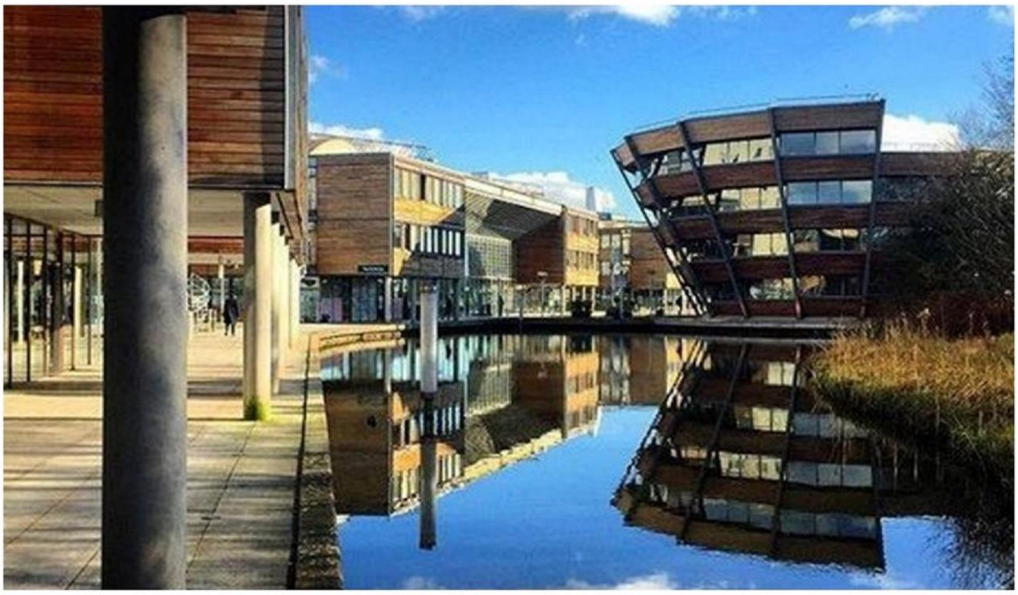

Once again thank you for your support on this project.

Please follow us on twitter for updates @HyperacusisJLA.

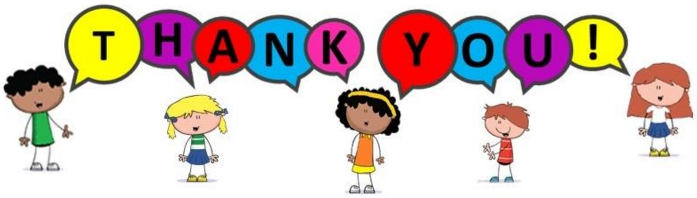

Privacy Notice

Here at the Hyperacusis James Lind Alliance Priority Setting Partnership (PSP) we take your privacy seriously. If you choose to send us your name and preferred contact details, we will only use this information for the purposes of contacting you to invite you to participate in future activities related to this PSP, which you might be interested in. There are no consequences to you if you do not want to provide your name and contact details.

The lawful basis for holding this data is that there is a legitimate interest for you to be contacted about future PSP activities. This helps the PSP ensure it listens to a variety of perspectives. The only people who have access to your name and contact details is the Administrator of this PSP and the research Lead. Your name and contact details are not linked to the survey in any way, and are not shared with anyone else. Your information is not subjected to any automatic processing or decision making.

Your name and contact details will be deleted at latest when the activities of this PSP close in August 2018. You have the right to see what information we hold about you, to correct it, and to ask us to delete it, at any time.

If you would like any further information or have any questions, please contact the PSP Administrator, Kathryn Fackrell on JLAhyperacusis@nottingham.ac.uk or look at our website: http://www.hearing.nihr.ac.uk/research/hyperacusis-PSP

Finished

Supplemental appendix 4: Categories and sub-categories with example questions

**Categories** *Sub-categories*	**Example question**
**Causes/Risk factors**	
Genetics	Are there genetic or epigenetic factor that impact the likelihood of getting hyperacusis?
Pregnancy	Can hyperacusis be caused by hormonal changes in pregnancy?
Physical or psychological	Is it triggered by worry about hearing a loud noise or can a loud noise in itself trigger the condition?
Nerve damage	Can muscular damage or wear and tear to jaw joints or neck cause hyperacusis?
Treatment	Does the insertion of cotton swabs deep inside the ear canal cause hyperacusis?
Infections	Does glue ear cause hyperacusis?
Diet/Exercise	Does diet affect hyperacusis?
Behaviour	Can hyperacusis be learned?
Exposure	Can exposure to loud sounds worsen or cause hyperacusis?
**Exacerbation**	Is there proof that overprotecting exacerbates hyperacusis?
**Classification**	Should hyperacusis be categorised differently based on its underlying causes rather than assuming they're all related?
**Prevalence**	What are the percentages of co morbidity across these 3 conditions (hyperacusis, hearing loss and tinnitus)?
**Natural history**	What is the natural progression/prognosis?
**Mechanisms**	What role, if any, does the cochlear feedback mechanism have in hyperacusis?
**Links with other conditions**	
Attention Deficit & Hyperactivity disorder	Is hyperacusis associated with other medical conditions/disabilities such as ADHD?
Autism	Is there a link between Autism and hyperacusis?
Dementia	Is hyperacusis or a type a hyperacusis a common experience in dementias?
Hearing	What is the relationship between hyperacusis and hearing loss?
Non-ear-related	Is there links between hyperacusis and chronic fatigue syndrome?

Other sensitivities	Do people with hyperacusis have other CNS symptoms ...e.g. sensitivity to bright light?
Personality	Does personality affect patients with hyperacusis?
Mental health	What role does depression and anxiety, and other mental health issues, play in how it affects people?
Trauma	Is it connected to head injury?
**Assessment**	What audiological test battery can be used by audiologists to detect hyperacusis and help monitor progress or regression in their patient's follow up consultation sessions?
**Management**	
Amplification	Can there be a hearing aid that has some kind of built-in equalizer so that certain frequencies can be lowered/adjusted before they reach ears?
Associated conditions	How can management of hyperacusis be reasonably adjusted for adults with intellectual disabilities?
Complementary/ alternative therapies	Does yoga improve hyperacusis?
Digital intervention	How useful /effective is online treatment for hyperacusis?
Effective treatment (general)	What is the efficacy of the current treatment strategies?
Medication	Can Selective Serotonin Reuptake Inhibitors help adults with hyperacusis?
Self-management	What can I do in my own time while I wait for the professional treatment to start, to speed up recovery?
Sound therapy	Are sound generators effective in the management of hyperacusis in children?
Surgical	How does a stapedectomy cause hyperacusis?
Tinnitus Retraining Therapy	Does TRT based treatment help hyperacusis in the long term?
Psychological therapy	Does cognitive behavioural therapy help manage underlying anxiety to further reduce hyperacusis in patients?
Dental	In what ways can we alter dental treatment to make the experience less painful for those suffering from hyperacusis?
**Prevention**	Can hyperacusis be prevented by warning labels in concerts, album covers, music apps, headphone packaging, etc.?
**Awareness**	How to roll out better education of the condition once diagnosed for families?
**Schools**	Should children with hyperacusis receive any additional support or accommodations at school?
**Service provision & evaluation**	Should there be a joint psychology/ audiology approach to managing hyperacusis?-
**Training**	Is specialty training available to otolaryngologists or audiologists regarding hyperacusis?

**Support**	Do those who experience Hyperacusis benefit from attending support groups?
