# Supplementary material for: Identifying and prioritising unanswered research questions for people with hyperacusis: James Lind Alliance Hyperacusis Priority Setting Partnership
Source: BMJ Open. 2019 Nov 21;9(11):e032178. doi: 10.1136/bmjopen-2019-032178 (PMC6886978; doi:10.1136/bmjopen-2019-032178)
Supplement: Author's manuscript [file bmjopen-2019-032178.draft_revisions.pdf]

BMJ Open is committed to open peer review. As part of this commitment we make the peer review history of every article we publish publicly available.

When an article is published we post the peer reviewers' comments and the authors' responses online. We also post the versions of the paper that were used during peer review. These are the versions that the peer review comments apply to.

The versions of the paper that follow are the versions that were submitted during the peer review process. They are not the versions of record or the final published versions. They should not be cited or distributed as the published version of this manuscript.

BMJ Open is an open access journal and the full, final, typeset and author-corrected version of record of the manuscript is available on our site with no access controls, subscription charges or pay-per-view fees (http://bmjopen.bmj.com).

If you have any questions on BMJ Open's open peer review process please email info.bmjopen@bmj.com

BMJ Open

Identifying and prioritising unanswered research questions for people with hyperacusis: the James Lind Alliance Hyperacusis Priority Setting Partnership

Journal:	*BMJ Open*
Manuscript ID	bmjopen-2019-032178
Article Type:	Research
Date Submitted by the Author:	06-Jun-2019
Complete List of Authors:	Fackrell, Kathryn; University of Nottingham, NIHR Nottingham Biomedical Research Centre; University of Nottingham, Hearing Sciences, Division of Clinical Neuroscience, School of medicine<br>Stratmann, Linda; University of Nottingham, NIHR Nottingham Biomedical Research Centre<br>Kennedy, Veronica; Bolton NHS Foundation Trust, Department of Paediatric Audiology<br>MacDonald, Carol; University of Stirling, Department of Psychology<br>Hodgson, Hilary; University of Nottingham, NIHR Nottingham Biomedical Research Centre<br>Wray, Nic; University of Nottingham, NIHR Nottingham Biomedical Research Centre<br>Farrell, Carolyn; University of Nottingham, NIHR Nottingham Biomedical Research Centre<br>Meadows, Mike; University of Nottingham, NIHR Nottingham Biomedical Research Centre<br>Sheldrake, Jacqueline; The Tinnitus and Hyperacusis Centre<br>Byrom, Peter; Peter Byrom Audiology Ltd<br>Baguley, David; University of Nottingham, NIHR Nottingham Biomedical Research Centre; Nottingham University Hospitals NHS Trust, Nottingham Audiology Services<br>Kentish, Rosie; University of Nottingham, NIHR Nottingham Biomedical Research Centre<br>Chapman, Sarah; Cochrane UK<br>Marriage, Josephine; Children's Hearing Evaluation & Amplification Resource<br>Phillips, JS; Norfolk and Norwich University Hospital NHS Trust, Department of Otolaryngology<br>Pollard, Tracey; Action on Hearing Loss<br>Henshaw, Helen; University of Nottingham, NIHR Nottingham Biomedical Research Centre; University of Nottingham, Hearing Sciences, Division of Clinical Neuroscience, School of medicine<br>Gronlund, Toto; James Lind Alliance, NIHR Evaluation, Trials and Studies Coordinating Centre<br>Hoare, Derek; University of Nottingham, NIHR Nottingham Biomedical Research Centre; University of Nottingham, Hearing Sciences, Division of Clinical Neuroscience, School of medicine
Keywords:	Hyperacusis, James Lind Alliance, Research, Management, Assessment

Identifying and prioritising unanswered research questions for people with hyperacusis: the James Lind Alliance Hyperacusis Priority Setting Partnership

Kathryn Fackrell[1,2,3], Linda Stratmann[1], Veronica Kennedy[4], Carol MacDonald[5], Hilary Hodgson[1], Nic Wray[1], Carolyn Farrell[1], Mike Meadows[1], Jacqueline Sheldrake[6], Peter Byrom[7], David M Baguley[1,2,8], Rosie Kentish[1], Sarah Chapman[9], Josephine Marriage[10], John Phillips[11], Tracey Pollard[12], Helen Henshaw[1,2], Toto Anne Gronlund[13], Derek J Hoare[1,2]

[1] National Institute for Health Research Nottingham Biomedical Research Centre, Nottingham, UK

[2] Hearing Sciences, Division of Clinical Neuroscience, School of medicine, University of Nottingham, Nottingham, UK

[3] National Institute for Health Research Evaluation, Trials and Studies Coordinating Centre, University of Southampton, Southampton, UK

[4] Department of Paediatric Audiology, Bolton NHS Foundation Trust, Bolton, UK

[5] Department of Psychology, University of Stirling, Stirling, UK

[6] The Tinnitus and Hyperacusis Centre, London, UK

[7] Peter Byrom Audiology Ltd, Sheffield, UK

[8] Nottingham Audiology Services, Nottingham University NHS Trust, Nottingham, UK

[9] Cochrane UK, Oxford, UK

[10] Children's Hearing Evaluation & Amplification Resource, London, UK

[11] Department of Otolaryngology, Norfolk and Norwich University Hospitals NHS Foundation Trust, Norwich, UK

[12] Action on Hearing Loss, London, UK

[13] James Lind Alliance, National Institute for Health Research Evaluation, Trials and Studies Coordinating Centre, Southampton, UK

Corresponding author: Kathryn Fackrell, NIHR Nottingham Biomedical Research Centre, Ropewalk House, 113 The Ropewalk, Nottingham, NG1 5DU, UK

Email: kathryn.fackrell@nottingham.ac.uk

For peer review only - http://bmjopen.bmj.com/site/about/guidelines.xhtml

ABSTRACT:

Objective

To determine research priorities in hyperacusis that key stakeholders agree are the most important.

Design/setting

A priority setting partnership using two international surveys, and a UK prioritisation workshop, adhering to the six-staged methodology outlined by the James Lind Alliance.

Participants

People with lived experience of hyperacusis, parents/carers, family and friends, educational professionals and healthcare professionals who support and/or treat adults and children who experience hyperacusis, including but not limited to surgeons, audiologists, psychologists, and hearing therapists.

Methods

The priority setting partnership was conducted from August 2017 to July 2018. An international identification survey asked respondents to submit any questions/uncertainties about hyperacusis. Uncertainties were categorised, refined, and rephrased into representative indicative questions using thematic analysis techniques. These questions were verified as "unanswered" through searches of current evidence. A second international survey asked respondents to vote for their top 10 priority questions. A shortlist of questions that represented votes from all stakeholder groups was prioritised into a top 10 at the final prioritisation workshop (UK).

Results

In the identification survey, 312 respondents submitted 2730 uncertainties. Of those uncertainties, 593 were removed as out of scope, and the remaining were refined into 85 indicative questions. None of the indicative questions had already been answered in research. The second survey collected votes from 327 respondents, which resulted in a shortlist of 28 representative questions for the final workshop. Consensus was reached on the top 10 priorities for future research, including identifying causes and underlying mechanisms, effective management, and training for healthcare professionals.

Conclusions

These priorities were identified and shaped by people with lived experience, parents/carers, and healthcare professionals, and as such are an essential resource for directing future research in hyperacusis. Researchers and funders should focus on addressing these priorities.

For peer review only - http://bmjopen.bmj.com/site/about/guidelines.xhtml

STRENGTHS AND WEAKNESSES OF THIS STUDY

1. This is the first time patients and healthcare professionals in hyperacusis have worked together to identify priorities for research
2. Used established James Lind Alliance methodology to systematically and transparently identify top research priorities
3. Survey responses from key stakeholders all over the world, which led to representative priorities for the UK, but with applicability internationally.
4. Some final indicative questions are broad and may be open to interpretation

For peer review only

For peer review only - http://bmjopen.bmj.com/site/about/guidelines.xhtml

INTRODUCTION

Hyperacusis is a hearing disorder involving an increased sensitivity or decreased tolerance to sound at levels that would not trouble most individuals. It differs from recruitment (a narrowing of the auditory dynamic range due to hearing loss), misophonia (an acquired aversive reaction to specific human generated sounds), and phonophobia (persistent, abnormal, and unwarranted fear of sound)[1–3]. For the person experiencing hyperacusis, everyday sounds of various loudness, such as the sound of the domestic appliances or electric hand dryers, can be uncomfortable or painful, and are perceived much louder and more intense than they are. This can be frightening and overwhelming and can cause anxiety and distress. Hyperacusis occurs as a primary complaint but is also commonly associated with other conditions including tinnitus, head trauma, depression, dementia, posttraumatic stress syndrome, and fibromyalgia [4]. Current estimates suggest that hyperacusis is experienced by 3.7% of children [5], and up to 9.2% of adults in a general population [6], with only 2% formally diagnosed by a physician[7]. Prevalence is particularly high in certain populations, for example, 65% of children and adults with Asperger's Syndrome or Autism Spectrum Disorder [8,9], and 95% of children and young adults with Williams syndrome experience hyperacusis [10,11].

Often chronic and disabling, hyperacusis can have a significant effect on daily life. For example, it can interfere with education, the ability to work, and/or participation in social and family life. In a recent cohort study involving 357 adult patients, 25 distinct domains of hyperacusis-associated problems were identified, including fear, avoidance behaviours, pain, activity limitations and impairments to quality of life such as reduced ability to work and socialise [12]. For children with hyperacusis, school environments (including lunch rooms and classrooms) can be challenging places, and strategies need to be in place to ensure that their wellbeing and educational needs are met [13]. People experiencing hyperacusis often become isolated and lose independence, avoiding situations where noise levels are outside their control. This not only affects the individual but also their family or carers. Many thousands of people experiencing hyperacusis or their family members turn to support and information provided online through Facebook, forums, and other social media [14].

Hyperacusis remains medically unexplained with no definitive diagnosis, aetiology, or cure [4]. For clinicians, the focus is on helping people manage their symptoms, not “cure” their hyperacusis, although no formal clinical practice guidance currently exists. The structure and provision of services for hyperacusis varies across the UK, with a variety of different medical specialties (including Ear, Nose and Throat, Audiology, Audiovestibular Medicine, Clinical Psychology) involved in the management of people experiencing hyperacusis. Clinical management in general includes non-standardised assessment using sound-based tolerance tests, and questionnaires to determine and measure the

For peer review only - http://bmjopen.bmj.com/site/about/guidelines.xhtml

specific effects hyperacusis has on an individual's life [15]. Approaches to treatment include sound therapy, counselling, and cognitive behaviour therapy (CBT) [16] but there is little or no evidence of their effectiveness. Indeed, despite its prevalence and clinical implications, hyperacusis research is in its infancy and there are many controversies in the field [17–19]. For example, only two randomised controlled trials specifically testing hyperacusis treatments have been conducted [16], and the only systematic review on hyperacusis examines prevalence in children and adolescents [20]. With such an open field, it is essential to identify and address research priorities that are immediately relevant and important to those affected by hyperacusis. The James Lind Alliance (JLA), a non-profit initiative hosted by the National Institute of Health Research (NIHR), provides the perfect platform to identify priorities that are relevant and potentially beneficial for all. The JLA guiding principles are to bring together patients, carers, and healthcare professionals in Priority Setting Partnerships (PSP) to identify and agree priorities for research. The JLA provides established step-by-step methodology to manage and complete a PSP, and ensures that all processes are accountable and transparent.

The aim of this study was (i) to identify the questions about hyperacusis that are important to people with lived experience, parents/carers, family, educational professionals and healthcare practitioners involved in the care of people who have hyperacusis and (ii) to prioritise these questions according to the relevance and importance to these groups to direct future research and funding in hyperacusis.

METHODS

The hyperacusis PSP took place over an 11-month period between August 2017 and July 2018. The six-stage methodology adhered to the PSP process prescribed by the JLA (Figure 1). Documentation produced for the PSP, including the protocol, posters and initial survey are published on the JLA website (http://www.jla.nihr.ac.uk/priority-setting-partnerships/hyperacusis/).

Stage 1. Establishing a working partnership

The research leads (KF & DH) from NIHR Nottingham Biomedical Research Centre (BRC), with support from our user organisation representative (LS), initiated the PSP and appointed a steering group to oversee and contribute to the PSP process. The steering group included people with lived experience of hyperacusis, a parent of a child living with hyperacusis, members of organisations who support those with hyperacusis, and healthcare professionals (audiologists, clinical psychologists, ENT and audiovestibular medicine representatives) with experience of working with and supporting those (adult and children) with hyperacusis (Supplemental appendix 1). The researchers (KF, DH, HHe) were allowed to attend the steering group meetings but did not participate in the prioritisation process. In order to reach a wide range of stakeholders, a small number of relevant partner organisations were

For peer review only - http://bmjopen.bmj.com/site/about/guidelines.xhtml

identified through Steering group members with established links (Supplemental appendix 2). A JLA adviser acted as neutral facilitator for the PSP, providing support and guidance throughout the whole PSP process, ensuring that there were equal contributions from all key stakeholders and that at all times the PSP was conducted in a fair and transparent way. The PSP coordinator and information specialist (KF) managed the surveys and data, communication, and performed the analysis and literature searches. The steering group directed and advised on all stages of the PSP, including the scope and relevant target stakeholders (Figure 1.).

Patient and Public Involvement

Patient and public involvement is a key component of the JLA process. People with lived experience and parents/carers of those with hyperacusis were included in the steering group for the PSP. As steering group members, they were actively involved in all aspects of the project, including co-producing all materials, undertaking thematic analysis on subsets of submitted uncertainties, and reviewing all documentation. The dissemination strategy, developed with the steering group, included engagement with members of the public through plain English summaries, charity magazine articles, and an Evidently Cochrane blog co-produced with our patient representatives (https://www.evidentlycochrane.net/hyperacusis-what-need-to-know/).

*** Insert Figure 1. ***

Stage 2. Consultation and agreeing scope

The steering group raised concerns over the numerous definitions associated with hyperacusis. Definitions in the literature include but are not limited to "*decreased sound tolerance and difficulty adjusting to sudden shifts in volume*" [21], "*a heightened awareness of sound*"[22] or "*hypersensitivity to sound*"[23], "*intolerance to everyday sounds that causes significant distress and impairment in social, occupational, recreational, and other day-to- day activities*"[24] and "*negative reactions following exposure to sound that would not evoke the same response in an average listener*"[25]. Patient representatives passionately felt that the definition needed to state that it was a hearing condition, and that a description of the experience had to be provided. Therefore, using evidence from a recent scoping review[16] and the experience of steering group members, we defined hyperacusis as "*a hearing disorder involving an increased sensitivity or decreased tolerance to sound at levels that would not trouble most individuals. For the person experiencing hyperacusis everyday sounds can be unpleasant, intense, frightening, painful, and overwhelming and can cause anxiety and distress.*"

For peer review only - http://bmjopen.bmj.com/site/about/guidelines.xhtml

All uncertainties related to hyperacusis for both adults and children were considered within scope. For example, the scope included uncertainties about the (i) causes, (ii) assessment, (iii) management (treatment, rehabilitation, self-management, interventions) of hyperacusis; (iv) prevention and education, (v) care and support; and (vi) healthcare services for hyperacusis. To be inclusive and ensure a representative range of hyperacusis uncertainties were identified, the steering group made the decision that the initial survey and interim prioritisation would be available internationally. However, due to funding restrictions it was agreed that the final prioritisation workshop would involve UK-based representatives only.

Stage 3. Identification of potential research questions

An online and paper version of the survey were co-produced with the steering group. The survey consisted of an explanation of the study and what was required, six open questions relating to each element of the scope (e.g. What question(s) about the *how hyperacusis is assessed and/or diagnosed* would you like to see answered by research?) with example questions (e.g. How do clinicians check for [xxx] in children?) and an open general question (Do you have any other questions that you feel are important for researchers to answer but do not fall in the areas above?). Participants were instructed that each question was optional and that they could write as much or as little as they liked for each. Participants were also asked to complete an optional demographic information section (stakeholder group, age, gender, country of residence, ethnic background, healthcare professionals' main profession, and any other hearing conditions). Participants answered the survey anonymously and were instructed to contact the study coordinator (KF) via a study-specific email address if they wanted to be involved in the next stages of the study.

A pilot phase was used with the steering group and researchers at NIHR Nottingham BRC (including a public and patient involvement manager) to check that the survey was written clearly, understandable, and easy to complete using the online survey software (www.surveymonkey.com). The text in the survey was refined to provide clarity on the number of questions that participants had to complete (i.e. each question was optional). The survey was circulated via steering group members, partner organisation contact lists, promotion in newsletters and national conferences, printed posters in clinics, and through dedicated social media channels. In addition to submitted uncertainties, we also searched for any research uncertainties highlighted in reviews of hyperacusis.

Stage 4. Refining research questions and identification of existing literature

The aim of this stage was to categorise and refine uncertainties, verify them as uncertainties and create a list of indicative questions for the next stage. A qualitative thematic analysis approach was taken to categorise and refine uncertainties, and generate a smaller number of representative

For peer review only - http://bmjopen.bmj.com/site/about/guidelines.xhtml

indicative questions. This involved (i) data familiarisation (reading and re-reading submissions), (ii) developing codes for uncertainties, (iii) identifying potential categories and sub-categories, and finally (iv) reviewing and refining final categories and sub-categories. Based on the categorisation, the uncertainties were rephrased into representative indicative research questions. The analysis was undertaken by the information specialist with input from steering group members throughout. The decision about whether a submission was out of scope was initially decided by the information specialist and subsequently reviewed by the steering group. Submissions were excluded based on criteria set by the steering group before the analysis was conducted. The criteria was any uncertainties (i) outside of scope (e.g. uncertainties about misophonia or recruitment alone), (ii) advice or information requests, and (iii) broad or ambiguous submissions. Because a previous PSP on tinnitus had ranked a research priority on the relationship between tinnitus and hyperacusis in their top 10 [26], the steering group decided that any submissions related to tinnitus would also be excluded. Where more than one uncertainty was identified in the same submission, the response was duplicated and assigned to the appropriate category. The final categories and refined questions with the original submitted uncertainties were reviewed by all steering group members to ensure that the final indicative questions were understandable and true representations of the original uncertainties. The steering group agreed the final list of indicative questions.

The verification process involved a review of current evidence for each indicative question to determine whether the questions were 'true uncertainties' or had already been answered by research. Given the broad scope, a comprehensive search approach was taken to ensure that all available evidence for indicative questions was identified. The Cochrane Ear, Nose and Throat Disorders Group Trials Register; the Cochrane Central Register of Controlled Trials (CENTRAL); PubMed; Embase; International Standard Randomised Controlled Trial Number (ISRCTN) registry; ClinicalTrials.gov; Google Scholar; and Google were searched from inception of each question to May 2018 for systematic reviews, past and current trials, scoping reviews, and clinical guidelines (including guidelines produced for hearing and tinnitus to check whether there was any evidence presented for hyperacusis) relating to the indicative questions. All evidence was recorded for each indicative question with links to journal papers. Indicative questions were only considered 'true uncertainties' where there was no evidence available, or was of low quality (i.e. likely further evidence would change the estimate of the effect), or where the available systematic reviews needed updating or indicated continuing uncertainties. The steering group reviewed all evidence and agreed a final list of questions which were included in the interim prioritisation survey.

For peer review only - http://bmjopen.bmj.com/site/about/guidelines.xhtml

Stage 5. Interim prioritisation

The purpose of this stage was to reduce the long list of questions to a shorter manageable by asking a wide range of people with lived experience of hyperacusis, and professionals to prioritise the indicative questions they considered most important. An online survey was created for this purpose using an interactive card-sorting software (www.optimalworkshop.com). The refined list of indicative questions was presented in a randomised sequence of cards for each participant on the screen. Participants were asked to read all the questions and select (drag and drop) the 10 questions that they thought were most important for research, in any order they wished (they were not asked to rank the questions). To help with the process of identifying their top 10 questions, an intermediate "questions of interest" box was included as a holding pen for questions they wished to consider. Participants were informed that they could swap and change questions from the boxes as often as they liked. Before taking part in the card sorting, participants were required to specify the stakeholder group(s) that applied to them. Once again, all survey responses were anonymous. Optional demographic information was collected (age, gender, healthcare professionals' main profession) and participants were invited to register their interest to attend the prioritisation workshop via email (Supplemental appendix 3). The survey was piloted with the steering group and researchers from NIHR Nottingham BRC to check that all instructions were clear and understandable, and to confirm the usability of the software. Instructions were refined to ensure clarity on the minimum number of questions to include in the "Top 10" box. The survey was circulated as an open invitation that was not restricted to those who had completed the first survey. The invitation was also sent to all people who had registered an interest in participating in the next stage following the initial survey, and circulated via steering group members, partner organisation contact lists, promotions in newsletters and at tinnitus support groups, printed posters in clinics, and through dedicated social media channels.

All indicative questions that were moved to the "Top 10" box were ranked based on the number of summed responses for each stakeholder group. For the analysis, weighted quantitative methods were used, in which the summed responses from professionals were weighted based on the number of responses from people with lived experience. This method was used, and agreed by the steering group, to ensure that the final indicative questions for the workshop were representative of all groups (i.e. of equal significance to votes from across all stakeholder groups). The results of the analysis were reviewed. The steering group also decided that if questions that captured under-represented groups (mental health, autism, dementia, learning difficulties) were not represented in the top set then these questions would be taken forward to the final workshop and the decision was made to take a top set of approximately 25-30 indicative questions to the workshop..

For peer review only - http://bmjopen.bmj.com/site/about/guidelines.xhtml

Stage 6. Final prioritisation workshop

A one-day workshop with representatives from each stakeholder group was held at the University of Nottingham in July 2018. The purpose of the workshop was to establish consensus on the top 10 research priorities for hyperacusis through a blend of whole group and sub-groups discussions facilitated by independent JLA advisers with no experience of hyperacusis. The workshop was chaired by the JLA adviser for this PSP (TAG). Remote participation to the final workshop was considered by the steering group but determined not feasible due to volume-control issues for representatives living with hyperacusis and small/large group discussion format. Therefore, only UK-based representatives were invited to participate in the final workshop. Healthcare and educational professionals, people with lived experience of hyperacusis, and parents of children living with hyperacusis were invited to participate via contact details submitted following the two surveys, organisation contact lists, and social media. Participants were selected with the aim of 50:50 representation for “lived experience” (including adults, children, and parents) and professionals. A small number of steering group members were encouraged to participate in the final workshop. Attending steering group members ensured all participants were supported with information on the PSP processes and any other support as required.

Using a modified nominal group process, participants were divided into three groups with mixed representatives from people with lived experience and professionals. Each group worked together initially discussing their own personal priorities. In the second round, cards with the indicative questions printed on them were laid out for the group to view, loosely ordered to reflect the discussion in the first round. The group were tasked to work together to rank the questions from most important to least. On the back of the cards were the results from the interim prioritisation survey to assist with decision-making, if needed. The rankings from each group were combined, and the membership of the groups was changed so that each group now had someone from one of the other two groups (again with mixed representatives in each). These new small groups were all presented the combined rankings from the previous round, and tasked to review and adjust the ranking to reflect the consensus of the group. The rankings from each group were again combined and finally, the whole group came together to discuss and adjust this combined ranking, until whole group consensus was reached on the top 10 priorities for hyperacusis.

RESULTS

Figure 2 shows a flow diagram of the study.

*** Insert Figure 2. ***

For peer review only - http://bmjopen.bmj.com/site/about/guidelines.xhtml

Demographics of initial survey respondents

Two-thousand, three-hundred and seventy uncertainties about hyperacusis were submitted, online and on paper, by 312 respondents, from October 2017 to January 2018. The number of uncertainties submitted per respondent ranged from 1 to 37, with the average number submitted per respondent being 10. Respondents included people with lived experience (58%), healthcare professionals from a range of disciplines (28%), parents/carers (7%), and family and friends (1%) of those with hyperacusis. A small number of educational professionals (1%) also submitted uncertainties on the school environment and children living with hyperacusis (Table 1). Several respondents also identified themselves as having an additional role, including respondents with lived experience of hyperacusis who also identified themselves as a parent (n=1), healthcare professional (n=1) or educational professional (n=1) (Table 1). Respondents were mainly residents in the United Kingdom (65%), America (13%) and Ireland (4%), with remaining residing in various countries in Europe, Asia and Africa. Demographics of the survey respondents are presented in Table 1. No additional questions were identified in the research literature that were not already covered by the uncertainties submitted.

*** Insert Table 1. ***

Refinement of research questions and identification of existing literature

Of the 2370 submitted uncertainties, 593 were removed as out of scope. The remaining 1777 uncertainties were subjected to thematic analysis and generated 15 categories (Figure 2). Each category grouped at least two uncertainties submitted by more than one respondent from the stakeholder groups. Sub-categories were generated for the larger categories (causes, management and links with other conditions) to facilitate the process of rephrasing uncertainties into representative indicative questions (Supplemental appendix 4). The grouped uncertainties were refined, and 85 indicative questions were formulated as representative of the original uncertainties. As a result of discussions, the steering group decided to leave some indicative questions as generic in terms of age (i.e. specifying children or adults). This would allow for researchers to address these from both angles and therefore increasing the number of possible priorities addressed. The existing literature did not provide any high quality evidence that addressed any of the indicative questions, therefore all 85 indicative questions were included in the interim prioritisation.

For peer review only - http://bmjopen.bmj.com/site/about/guidelines.xhtml

Interim priorities determined from second survey

The interim prioritisation survey opened in May 2018 and closed in July 2018. Three-hundred and twenty-seven respondents voted for their top 10 indicative research questions. Respondents were people with lived experience (67%), healthcare professionals (25%) and then a small number of parents/carers (6%), family and friends (1%), and educational professionals (1%). Demographics of the survey respondents are presented in Table 1. As expected, there was some imbalance in the priorities of different stakeholder groups. However, the weighted ranked scores for all 85 questions were reviewed by the steering group and the top 27 indicative questions were identified as representative of the votes received by all stakeholder groups. For example, 18 of the top 25 questions voted for by people with lived experience were included in the top 27. It was agreed, as per the *a priori* criteria set by the steering group, that the top 27 indicative questions, and an additional indicative question on dementia, were all priorities (Table 2).

*** Insert Table 2. ***

Top 10 priorities

The one-day workshop held in July 2018 included 21 participants, representing people with lived experience of hyperacusis (n = 6) (including two teachers), parents of children living with hyperacusis (n = 5), and healthcare professionals (n = 10) from a range of disciplines that care for and support those with hyperacusis (including audiologists, a paediatric clinical psychologist, clinical psychologists, and an audiovestibular physician). Following this workshop, consensus was reached on a top 10 research priorities for hyperacusis (Table 3). These questions cover a broad range of topics in hyperacusis including causes, mechanisms, prevalence, management and treatment, and health-care provider knowledge and training.

Publicity

The top 10 priorities were disseminated to all participants involved in the process via a contact email address or social media and shared by steering group members and partner organisations through their contact lists. The top 10 priorities were unveiled at the British Academy of Audiology national conference in November 2018 [27], published in audiology and charity magazine articles [28,29] and in a published letter in a scientific journal [30]. The list of original submitted uncertainties, the 85 indicative questions, and the final list of priorities taken to the workshop have been made available

For peer review only - http://bmjopen.bmj.com/site/about/guidelines.xhtml

on and promoted through the JLA website (http://www.jla.nihr.ac.uk/priority-setting-partnerships/hyperacusis/).

*** Insert Table 3. ***

DISCUSSION

The hyperacusis PSP has successfully brought together people with lived experience of hyperacusis and healthcare professionals in hyperacusis to identify and agree priorities for research. These priorities were therefore identified and shaped by people with lived experience, parents/carers and healthcare professionals. Although this process did not identify radically different questions, it did highlight the sheer number of unanswered questions about hyperacusis. The top 10 priorities reflect the lack of evidence and guidance on the assessment and management of hyperacusis, including the need for training in hyperacusis. These priorities highlight the need for research to focus on the underlying mechanisms and types of hyperacusis, and prevalence of hyperacusis in specific populations. The list of priorities identified here will therefore be of interest to researchers and funders of hearing/ health research, and will hopefully direct future research and funding calls.

There are many notable strengths to this study. Firstly, people with lived experience and parents/carers were involved throughout the process, from the initial planning for the JLA with our user organisation representative, planning and overseeing the project as members of the steering group, and participation in both surveys worldwide, and in the final workshop. All of this enabled people with lived experience and parents/carers to have the opportunity for a voice and to influence the future priorities for research on hyperacusis. Secondly, this PSP also took an inclusive approach to gathering the uncertainties and interim voting by having two international online surveys, and although the final consensus on the priorities was reached with UK representatives only, the top 10 priorities, and the longer list of priorities, have international relevance as they represent unanswered questions submitted by people with lived experience, parents/carers and healthcare professionals from around the world. Whilst it is possible that some health service provision and evaluation questions are less relevant in some countries, the remaining indicative questions collected in this PSP should be considered unanswered priorities for all. Thirdly, although more people with lived experience engaged with both surveys than healthcare professionals, by using weighted ranking, the top 10 reflected the mixed priorities from all stakeholder groups, of which five were in the top 10 for

For peer review only - http://bmjopen.bmj.com/site/about/guidelines.xhtml

people with lived experience and parents/carers during the interim prioritisation and six were in the top 10 for professionals.

There were some challenges with the PSP process. One challenge was interpreting the submissions that were more narrative stories than specific research questions. Due to the nature of the PSP, many submissions were asking more than one question within a narrative story, especially submissions from people experiencing hyperacusis who until this point had not been asked their point of view. To ensure that all vital information and possible questions from the narratives were identified and recorded, the information specialist worked closely with steering group members to interpret the narratives. Another challenge arose in planning the JLA final face-to-face workshop; people who experience hyperacusis find that travelling and participating in large and small group discussions can lead to pain, discomfort, or distress. In order to overcome any foreseeable problems with recruiting people with lived experience, different options were discussed by the steering group. Remote participation was suggested as a potential avenue for people to take part in the workshop. However, it was decided from experience of remote steering group meetings, and advice sought from the JLA, that online discussion facilities such as Skype or teleconferences would not overcome the problems with discomfort, would be physically and mentally taxing to participants, and could impede the discussion process. The venue for the workshop was discussed. It was decided that the workshop would take place at the University of Nottingham during the summer period when there are no students on campus as this would reduce the number of potential problems with loud noises. The facilities used for the workshop were quiet rooms with limited external sounds. Participants were given the option to travel the day before the workshop to allow time to recover from travelling. All facilitators and participants were aware of potential problems that could occur and a dedicated quiet room was available for anyone to go to for a break. The lead researchers were on hand to offer any additional support to participants. Therefore, although a relatively small number of people with lived experience participated in the final workshop (six people with lived experience and five parents of children with hyperacusis), it did not impede the discussions or consensus as there was balanced representation from each stakeholder group. Also, the results from interim prioritisation, where a large number people experiencing hyperacusis voted, informed the discussions.

Many of the priorities highlighted by this PSP clearly mark areas that are less well researched in hyperacusis. For example, in 2014, a two-part review of hyperacusis research [17,18] highlighted key areas in hyperacusis that were seen as important to progress, including but not limited to, properly defining and subtyping hyperacusis, identifying and understanding mechanisms and the role of the brain, and evaluating current treatments (particularly sound therapy and drugs). These topics also appeared in reviews published in 2017 [16] and 2018 [19]. These more recent reviews also reported a

For peer review only - http://bmjopen.bmj.com/site/about/guidelines.xhtml

need for up-to-date prevalence figures and valid measurement tools. All these topics reflect the 28 priority questions identified in our interim prioritisation survey. This would suggest that the majority of recently published research in hyperacusis has not necessarily been addressing the areas of concern. A search of Google Scholar revealed that the most recent publications for hyperacusis have focused on basic research questions around causes of hyperacusis [31–33], or on research investigating hyperacusis and the workplace [34]. The only relevant ongoing trial is investigating "Exposure Therapy for Auditory Sensitivity in Autism" (ClinicalTrials.gov Identifier: NCT03206996). Although this research is important, it is somewhat disheartening to observe that the majority of the areas highlighted by a review in 2014 still remain an issue now. This prioritisation exercise highlighted the need for research to have a much greater focus on clinical needs (management approaches) and effective treatments, as well as aspects of underlying mechanisms and subtyping. We hope our results encourage researchers to focus on addressing these priorities with high quality research studies.

This PSP highlighted a potential challenge within current healthcare for hyperacusis. A larger number of patients engaged in each of the surveys than healthcare professionals. This could be a reflection of our current healthcare system, in which only a small number of healthcare professionals have experience in such a specialised subject as hyperacusis. Moreover, it was apparent from the list of priorities that identifying the essential training needs of healthcare professionals for each clinical speciality involved with hyperacusis is a priority. Furthermore, the number of out of scope uncertainties asking for information on hyperacusis and care and support further highlighted this problem and a clear need for greater awareness of hyperacusis. Current knowledge and awareness of hyperacusis in the UK is sadly lacking. Therefore, informational resources are needed to raise awareness of hyperacusis and provide guidance on seeking care and support. However, in order to provide this information, first service evaluations need to be conducted to provide a clear picture of the current position of hyperacusis care and identify the areas of need.

Conclusion

Hyperacusis is a topic of growing interest. Here we have identified a set of priority questions that reflect what people with lived experience, parents/carers, and healthcare professionals, want answered by research. As such researchers and funders should focus on addressing these priorities.

For peer review only - http://bmjopen.bmj.com/site/about/guidelines.xhtml

ACKNOWLEDGEMENTS

The authors wish to acknowledge the people with lived experience of hyperacusis, parents of children living with hyperacusis, families and friends, carers and educational and healthcare professionals who submitted responses to the international surveys. In addition, they would like to thank the partner organisations (Supplemental appendix 2) who supported and promoted this work, Sandra Smith for checking the literature searches and providing support at the final prioritisation workshop, the JLA for support and guidance throughout the process and all attendees and JLA facilitators (Katherine Cowan, Sheela Upadhyaya, and Toto Anne Gronlund) at the final workshop who worked tirelessly to achieve a consensus on the top 10 research questions.

FUNDING

This work was supported through grants from the British Society of Audiology and Action on Hearing Loss. The Partnership was supported by the National Institute for Health Research (NIHR) Nottingham Biomedical Research Centre (BRC).

DISCLAIMER

KF is funded by the National Institute for Health Research (NIHR Post-Doctoral Fellowship, Dr Kathryn Fackrell, PDF-2018-11-ST2-003). HHe is funded by the National Institute for Health Research (NIHR Career Development Fellowship, Dr Helen Henshaw, CDF-2018-11-st2-016). DJH is funded by the National Institute for Health Research (NIHR) Biomedical Research Centre programme. However the views expressed in this publication are those of the author(s) and not necessarily those of the NHS, the National Institute for Health Research or the Department of Health and Social Care.

COMPETING INTERESTS

DJH is vice-chair of the British Society of Audiology. KF is a member of the British Society of Audiology Tinnitus and Hyperacusis special interest group. DMB is president of the British Tinnitus Association. NW is an employee of the British Tinnitus Association.

CONTRIBUTIONS

KF and DH made the initial application to the JLA for a hyperacusis PSP. KF organised steering group meetings and teleconferences, contacted and enrolled all partner organisations, designed the surveys,

For peer review only

For peer review only - http://bmjopen.bmj.com/site/about/guidelines.xhtml

484 liaised with organisations throughout the PSP, promoted the PSP through a dedicated Twitter account, conducted thematic analysis and literature reviews, organised and enrolled participants for the final workshop, and drafted the final manuscript. DH organised venues and facilities for meetings and final workshop, drafted and circulated minutes of every meeting and teleconference, conducted initial thematic analysis and analysis on the interim prioritisation survey data. All authors contributed to the information presented in the surveys and promotions, conducted thematic analysis on a subset of submitted uncertainties and refined and rephrased the 85 indicative questions. All authors read and approved the final manuscript for submission.

PATIENT CONSENT

Not required.

ETHICS APPROVAL

This work did not require ethics approval as per the JLA guidance and guidance published by the NHS National Patient Safety Agency National Research Ethics Service.

PROVENANCE AND PEER REVIEW

Not commissioned; externally peer reviewed.

DATA SHARING STATEMENT

Supplementary data including all submitted original research uncertainties can be found on the JLA website at http://www.jla.nihr.ac.uk/priority-setting-partnerships/hyperacusis/

For peer review only

For peer review only - http://bmjopen.bmj.com/site/about/guidelines.xhtml

REFERENCES

1 McFerran D. Misophonia and Phonophobia. In: Baguley DM, Fagelson M, eds. *Tinnitus: clinical and research perspectives.* San Diego: : Plural Publishing Inc 2016. 245–60.

2 Kumar S, Tansley-Hancock O, Sedley W, *et al.* The Brain Basis for Misophonia. *Curr Biol* 2017;27:527–33.

3 Moore B. *Cochlear Hearing Loss: Physiological, Psychological and Technical Issues.* 2nd ed. Chichester, UK: : Wiley 2007.

4 Baguley DM. Hyperacusis. *J R Soc Med* 2003;96:582–5.

5 Hall AJ, Humphriss R, Baguley DM, *et al.* Prevalence and risk factors for reduced sound tolerance (hyperacusis) in children. *Int J Audiol* 2016;55:135–41.

6 Andersson G, Lindvall N, Hursti T, *et al.* Hypersensitivity to sound (hyperacusis): a prevalence study conducted via the Internet and post. *Int J Audiol* 2002;41:545–54.

7 Paulin J, Andersson L, Nordin S. Characteristics of hyperacusis in the general population. *Noise Heal* 2016;18:178–84.

8 Danesh AA, Lang D, Kaf W, *et al.* Tinnitus and hyperacusis in autism spectrum disorders with emphasis on high functioning individuals diagnosed with Asperger's Syndrome. *Int J Pediatr Otorhinolaryngol* 2015;79:1683–8.

9 Khalfa S, Bruneau N, Rogé B, *et al.* Increased perception of loudness in autism. *Hear Res* 2004;198:87–92.

10 Baguley DM, McFerran D. Hyperacusis and Disorders of Loudness Perception. In: Moller A, Langguth B, DeRidder D, *et al.*, eds. *Textbook of Tinnitus*. New York: : Springer 2011. 13–24.

11 Klein B, Mervis C. Contrasting patterns of cognitive abilities of 9-and 10-year-olds with William's syndrome or Down syndrome. *Dev Neuropsychol* 1999;16:177–96.

12 Fackrell K, Sereda M, Sheldrake J, *et al.* Why is Hyperacusis a Problem? A Qualitative Analysis of Problems Reported by Patients and Research Volunteers. In: *British Academy of Audiology's 14th Annual Conference*. Bournemouth, UK: 2017.

13 Myne S, Kennedy V. Hyperacusis in children: A clinical profile. *Int J Pediatr Otorhinolaryngol* 2018;107:80–5.

14 Fackrell K, Sereda M, Sheldrake J, *et al.* Untangling the relationship between tinnitus and hyperacusis: What are the problems reported by patients with a primary complaint of hyperacusis. In: *International Tinnitus Research Initiative Conference*. Regensburg, Germany: 2018.

15 Fackrell K, Hoare DJ. Scales and questionnaires for decreased sound tolerance. In: Baguley DM, Fagelson M, eds. *Hyperacusis and Disorders of Sound Intolerance: Clinical and Research Perspectives.* San Diego, CA: : Plural Publishing Inc 2018.

16 Fackrell K, Potgieter I, Shekhawat GS, *et al.* Clinical Interventions for Hyperacusis in Adults : A Scoping Review to Assess the Current Position and Determine Priorities for Research. *Biomed Res Int* 2017;2017.

17 Tyler RS, Pienkowski M, Roncancio ER, *et al.* A Review of Hyperacusis and Future Directions: Part I. Definitions and Manifestations. *Am J Audiol* 2014;23:402–419.

For peer review only - http://bmjopen.bmj.com/site/about/guidelines.xhtml

18 Pienkowski M, Tyler RS, Roncancio ER, *et al.* A Review of Hyperacusis and Future Directions: Part II. Measurement, Mechanisms, and Treatment. *Am J Audiol* 2014;23:420–436.

19 Baguley D, Hoare D. Hyperacusis: major research questions. *HNO* 2018;66:358–63.

20 Rosing SN, Schmidt JH, Wedderkopp N, *et al.* Prevalence of tinnitus and hyperacusis in children and adolescents: a systematic review. *BMJ Open* 2016;6:e010596.

21 Attri D, Nagarkar AN. Resolution of hyperacusis associated with depression, following lithium administration and directive counselling. *J Laryngol Otol* 2010;124:919–21.

22 Phillips D, Carr M. Disturbances of loudness perception. *J Am Acad Audiol* 1998;9:371–9.

23 Suchova L. Tinnitus retraining therapy--the experiences in Slovakia. *Bratisl Lek Listy* 2005;106:79–82.

24 Aazh H, Moore BCJ, Lammaing K, *et al.* Tinnitus and hyperacusis therapy in a UK National Health Service audiology department: Patients' evaluations of the effectiveness of treatments. *Int J Audiol* 2016;2027:1–9.

25 Jastreboff PJ, Jastreboff MM. Treatments for decreased sound tolerance (hyperacusis and misophonia). *Semin Hear* 2014;35:105–20.

26 Hall DA, Mohamad N, Firkins L, *et al.* Identifying and prioritizing unmet research questions for people with tinnitus: the James Lind Alliance Tinnitus Priority Setting Partnership. *Clin Investig (Lond)* 2013;3:21–8.

27 Fackrell K on behalf of the hyperacusis PSP steering group. Prioritising hyperacusis: The Top 10 research priorities. In: *British Academy of Audiology's 15th Annual Conference*. Liverpool, UK: 2018.

28 Hoare D, Fackrell K. Collaborating with patients on research priorities in hyperacusis: the James Lind Alliance project. *ENT News* 2018.

29 Fackrell K on behalf of the hyperacusis PSP steering group. Prioritising hyperacusis. *Quiet, Br Tinnitus Assoc* 2019.

30 Fackrell K, Stratmann L, Gronlund TA, *et al.* Top ten hyperacusis research priorities in the UK. *Lancet* 2019;393:404–5.

31 Kompis M, Hohl A, Seifert E, *et al.* Voluntary increase of acoustic middle ear impedances with simultaneous sound attenuation associated with mild hyperacusis (VIMH). *Acta Otolaryngol* 2019;:1–6.

32 Farahani S, Nasirinezhad F, Danyali S, *et al.* Does 5, 7-Dihydroxytryptamine injection into nucleus accumbens cause hyperacusis? *Neurosci Lett* Published Online First: 7 April 2019.

33 Silverstein H, Smith J, Kellermeyer B. Stapes hypermobility as a possible cause of hyperacusis. *Am J Otolaryngol* 2019;40:247–52.

34 Paulin J, Nordin M, Nyback M-H, *et al.* Associations between hyperacusis and psychosocial work factors in the general population. *Int Arch Occup Environ Health* 2019;92:59–65.

For peer review only

For peer review only - http://bmjopen.bmj.com/site/about/guidelines.xhtml

TABLES

Table 1. Demographics of respondents to the identification survey and interim prioritisation survey

Characteristics	Identification	Interim prioritisation
	n (dual role)	n (dual role)
**Role**		
Person with hyperacusis	181	218
Tinnitus	74	—
Hearing loss	36	—
Tinnitus and hearing loss	66	—
Parent of a child with hyperacusis	19 (2)	14 (4)
Carer of someone with hyperacusis	2 (1)	4 (5)
Friend or family member of someone with hyperacusis	3	4 (7)
Education professionals (e.g school teacher, lecturer)	3 (3)	2 (7)
Healthcare professional	87 (1)	82 (11)
Audiologist	43	38
Paediatric audiologist	7	2
ENT	2	6
Audiovestibular Physician	11	7
Clinical scientist	3	5
Hearing therapist	9	3
Speech-language pathologist	—	2
Speech and Language Therapist	—	1
Clinical Psychologist	4	
Psychiatrist	1	1
Clinical Hypnotherapist	1	—
Intensive care physician	—	1
GP	—	1
Paediatrician	1	2
Nurse	—	3
Dentist	—	1
Chiropractor and nutritionist	1	—
Other	8	3
Not specified	9	—
**Age**		
Under 10	0	4
10 - 20	7	11
21 - 30	19	30
31 - 40	40	49
41 - 50	67	37
51 - 60	85	59
61 - 70	57	42
71 - 80	16	19
80+	1	2
Not specified	20	74

For peer review only

For peer review only - http://bmjopen.bmj.com/site/about/guidelines.xhtml

**Sex**		
Female	194	95
Male	103	152
I would describe it in another way	—	2
Not specified	5	78
**Ethnicity**		
White	276	—
Asian	8	—
Mixed/ multiple ethnic group	4	—
Arab	1	—
Black	1	—
Prefer not to say	20	—
Other (not specified)	5	—
**Country of residence**		
United Kingdom (UK)	203	—
United States of America (USA)	42	—
Ireland	11	—
Australia	6	—
Canada	6	—
New Zealand	4	—
Sweden	4	—
Netherlands	3	—
Germany	2	—
South Africa	2	—
Austria	1	—
Belgium	1	—
Brazil	1	—
Denmark	1	—
Finland	1	—
France	1	—
Greece	1	—
Portugal	1	—
Philippines	1	—
Saudi Arabia	1	—
Switzerland	1	—

For peer review only

583 **Table 2. The top 28 ranked questions from interim prioritisation survey**

Research Question	Mean	Person with hyperacusis	Parents/ Carers/ family	Prof~
What is the most effective treatment approach for hyperacusis in adults?	49	60	6	80
Which treatment approaches are most effective for different types or severities of hyperacusis?	45	51	6	77
Does avoidance of sound improve hyperacusis or make it worse?	40	60	8	51
Is hyperacusis related to physical changes in the ear or brain?	39	69	3	46
Is hyperacusis due to physical or psychological issues or is it a combination of both?	38	41	7	65
Which psychological therapy (e.g. counselling, Cognitive Behavioural Therapy, mindfulness) is most effective for hyperacusis?	37	18	7	85
What are the 'red flags' for serious underlying conditions in hyperacusis?	34	28	7	68
Which criteria should be met to diagnose hyperacusis in adults/children?	34	25	5	71
What area(s) of the brain and patterns of activity is/are associated with hyperacusis?	34	51	4	46
What is the most effective treatment approach for hyperacusis in children?	33	10	7	83
What are the risk factors for developing hyperacusis or making it worse?	31	37	3	54
What is the best way of using sound in therapy for hyperacusis?	31	20	2	71
What is the essential knowledge/training required for health professionals to appropriately refer or effectively manage hyperacusis?	30	27	5	57
What management approach for hyperacusis is most effective for adults/children with autism?	29	8	5	74
Is there an association between hyperacusis and other ear-related conditions (e.g. superior canal dehiscence syndrome, Meniere's, Waardenburg syndrome, vertigo, vestibular migraines)?	27	36	3	43
What is the best way to differentiate hyperacusis from other hearing conditions (e.g. recruitment, misophonia, Meniere's, tinnitus)?	27	30	3	48
Which self-help interventions are effective for hyperacusis?	27	40	7	34
Is hyperacusis linked to other sensitivities/conditions?	26	34	11	34
Does nerve damage cause the pain associated with hyperacusis?	25	60	5	11
What is the prevalence of hyperacusis in a general population and other specific populations (e.g. people	25	17	3	54

For peer review only - http://bmjopen.bmj.com/site/about/guidelines.xhtml

with autism, mental health issues, learning disabilities, hearing loss)?				
Which drugs are effective for hyperacusis?	25	45	3	26
Can noise exposure cause hyperacusis (or make it worse)?	25	59	6	9
Which interventions in a school setting are useful for children with hyperacusis?	24	11	7	54
Would restoring hearing (e.g. regenerating nerve cells) improve hyperacusis?	22	56	5	6
Are there different meaningful types of hyperacusis?	22	23	3	40
What is the relationship between mental health and hyperacusis?	22	20	6	40
What care is most effective for recent onset/acute hyperacusis?	22	30	1	34
What is the association between hyperacusis and dementia?*	8	16	0	9

Prof = healthcare and educational professionals. ~weighted professionals score. *Additional question on dementia with lower ranking included at final workshop as per *a priori* criteria set by steering group.

For peer review only

For peer review only - http://bmjopen.bmj.com/site/about/guidelines.xhtml

Table 3. Hyperacusis Priority Setting Partnership top ten priorities for future research

Priority	Research Question
1	What is the most effective treatment approach for hyperacusis in children?
2	What is the prevalence of hyperacusis in a general population and other specific populations (e.g. people with autism, mental health issues, learning disabilities, hearing loss)?
3	Are there different meaningful types of hyperacusis?
4	What is the essential knowledge/training required for health professionals to appropriately refer or effectively manage hyperacusis?
5	Which treatment approaches are most effective for different types or severities of hyperacusis?
6	Is hyperacusis due to physical or psychological issues or is it a combination of both?
7	Which psychological therapy (e.g. counselling, Cognitive Behavioural Therapy, mindfulness) is most effective for hyperacusis?
8	What management approach for hyperacusis is most effective for adults/children with autism?
9	What is the best way of using sound in therapy for hyperacusis?
10	Which self-help interventions are effective for hyperacusis?

For peer review only

For peer review only - http://bmjopen.bmj.com/site/about/guidelines.xhtml

FIGURE LEGENDS

Figure 1. The six-stage methodological process for the Hyperacusis Priority Setting Partnership

Figure 2. Flow diagram of prioritisation process from submitted research questions to final prioritisation. * One addtional question on dementia with lower ranking was included at final workshop as per *a priori* criteria set by steering group.

SUPPLEMENTAL MATERIAL

Supplemental appendix 1. Steering Group of the Hyperacusis Priority Setting Partnership

Supplemental appendix 2: Partner organisations

Supplemental appendix 3: Interim prioritisation survey

Supplemental appendix 4: Categories and sub-categories with example questions

For peer review only - http://bmjopen.bmj.com/site/about/guidelines.xhtml

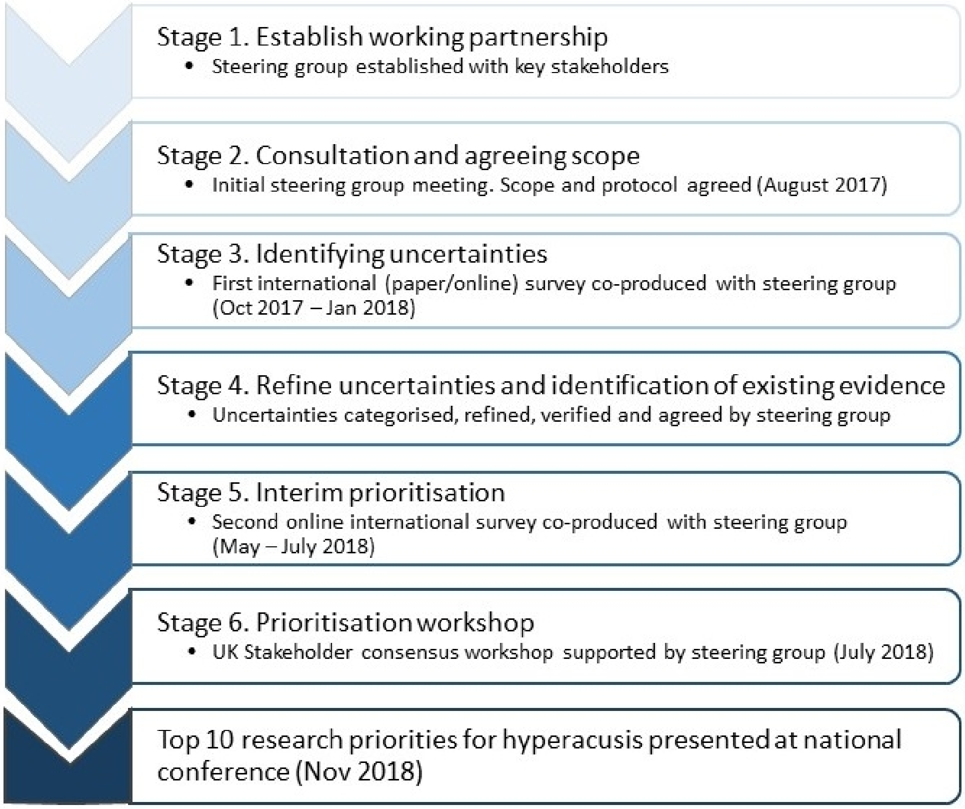

Figure 1. The six-stage methodological process for the Hyperacusis Priority Setting Partnership

182x154mm (96 x 96 DPI)

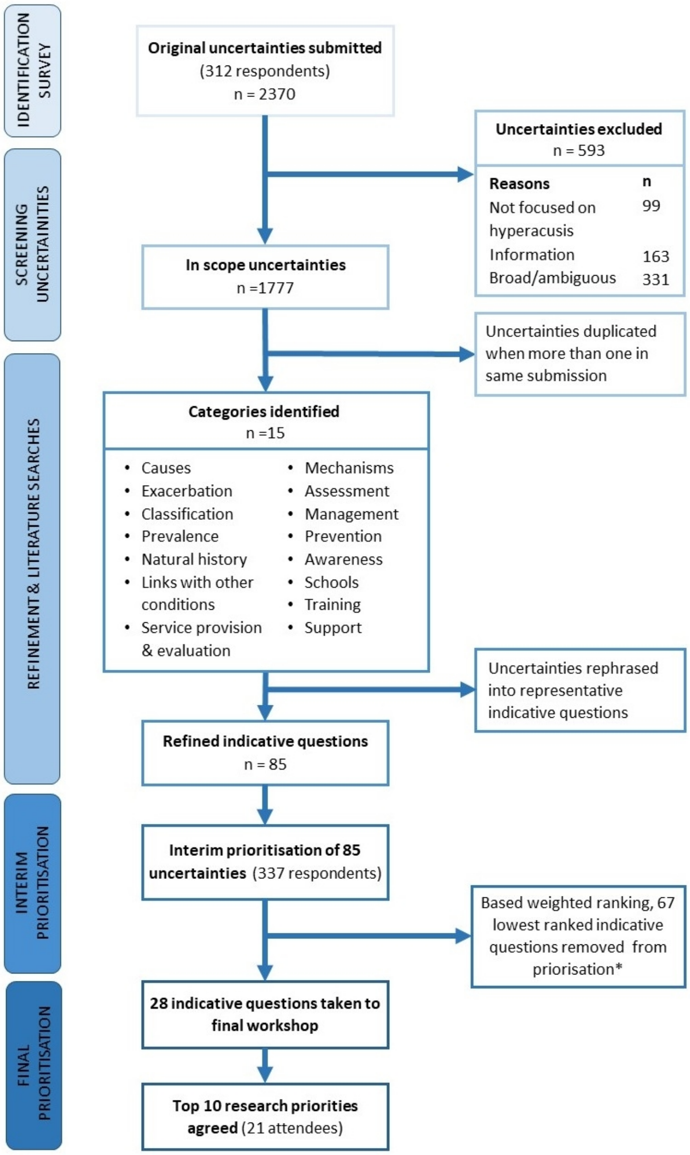

Figure 2. Flow diagram of prioritisation process from submitted research questions to final prioritisation.
* One addtional question on dementia with lower ranking was included at final workshop as per a priori criteria set by steering group.

233x344mm (96 x 96 DPI)

SUPPLEMENTAL MATERIALS

Supplemental appendix 1. Steering Group of the Hyperacusis Priority Setting Partnership.

Steering group member	Role
**James Lind Alliance support and guidance**	
Toto Anne Gronlund	Independent chair, representing James Lind Alliance
Katherine Cowan	Final workshop Facilitator, The James Lind Alliance
Sheela Upadhyaya	Final workshop Facilitator, The James Lind Alliance
**Non-voting members**	
Kathryn Fackrell	Study coordinator and Information specialist, Researcher, University of Nottingham
Derek Hoare	Researcher, University of Nottingham
Helen Henshaw	Researcher, University of Nottingham
**Patient representatives**	
Linda Stratmann	User organisation, founder and co-moderator of Facebook group Hyperacusis Support & Research, and person with lived experience
Tracey Pollard	Action on Hearing Loss patient representative
Nic Wray	Person with lived experience
Carolyn Farrell	Person with lived experience
Mike Meadows	Person with lived experience
Hilary Hodgson	Parent
Sarah Chapman	Cochrane UK Knowledge Broker
**Clinical representatives**	
Carol MacDonald	CBT psychotherapist and Clinical Psychology Lecturer
David Baguley	Clinical Scientist (audiology)
Jacqueline Sheldrake	Audiologist
John Phillips	ENT surgeon
Josephine Marriage	Paediatric audiologist
Peter Byrom	Audiologist
Rosie Kentish	Clinical Psychologist
Veronica Kennedy	Audiovestibular Physician

For peer review only

Supplemental appendix 2: Partner organisations

Charity organisations

Action on Hearing Loss

Space 4 Autism

Professional organisations

British Society of Audiology

British Association of Audiovestibular Physicians

National Institute of Health Research Nottingham Biomedical Research Centre

Social media outlets

Facebook group Hyperacusis Support & Research

National Institute of Health Research Nottingham Biomedical Research Centre Facebook

Study-dedicated twitter account (@HyperacusisJLA)

For peer review only

Supplemental appendix 3: Interim prioritisation survey

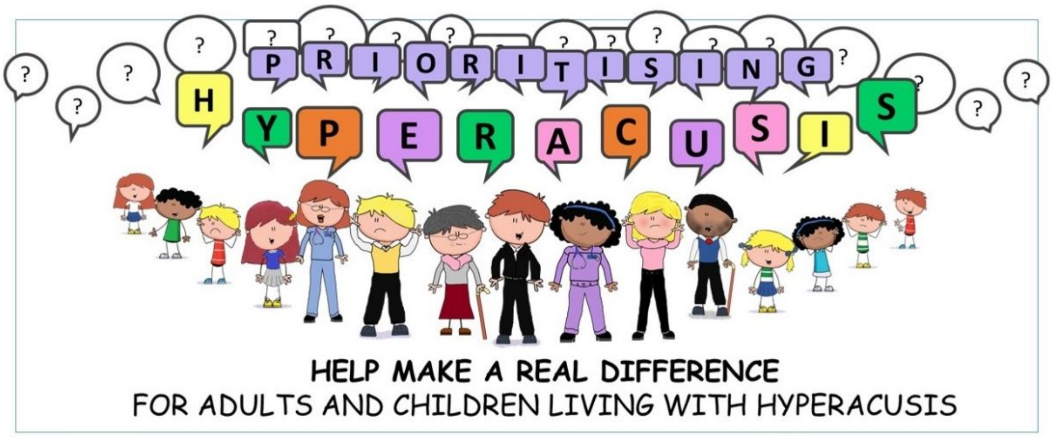

Welcome to this Hyperacusis James Lind Alliance Priority Setting Partnership survey.

Thank you for agreeing to participate!

What is hyperacusis?

Hyperacusis is a hearing disorder involving an increased sensitivity or decreased tolerance to everyday sound at levels that would not trouble most individuals. For the person experiencing hyperacusis everyday sounds can be unpleasant, intense, frightening, painful, and overwhelming and can cause anxiety affecting their quality of life.

We need you to vote for your Top 10 research questions about hyperacusis

We want to identify the Top 10 questions that research should target. Our first survey asked for people with lived experience of hyperacusis, parents, carers, teachers, members of the public and healthcare professionals who work with and support those who experience hyperacusis to submit any questions they had about hyperacusis.

In this survey you have the opportunity to choose 10 "unanswered" questions about hyperacusis that you think are the most important for research to target. Anyone with experience of hyperacusis (except researchers) can complete this survey. You do not have to have taken part in any previous studies.

The survey should not take longer than 15 minutes to complete.

Why do this?

Your responses will help us and other organisations to target research and funding for hyperacusis. This really is a unique opportunity to have your say and make sure that research addresses questions that matter to you and others like you.

Find out how on the next page...

For peer review only

Continue

Thank you for agreeing to complete this survey. Before we begin…

We just need to know your experience with hyperacusis to make sure that we understand the voting patterns by a wide range of people with different experiences. Anyone with experience of hyperacusis can complete this survey!

Which of the following best describes you?

Please select all the groups that apply to you. For example, if you are a healthcare professional and a parent of a child with hyperacusis then select both.

- ☐ Person with hyperacusis currently or in past
- ☐ Carer of someone with hyperacusis
- ☐ Parent of a child with hyperacusis
- ☐ Family or friend of someone with hyperacusis
- ☐ Educational professional, e.g. school teacher, lecturer
- ☐ Healthcare professional
- ☐ Other

Continue

For peer review only

Instructions

On the left of the screen in a list of "unanswered" research questions about hyperacusis.

On the right of screen are two category boxes ("questions of interest" and "Top 10"). The questions on the left of the screen are designed for you to move them (drag and drop them using mouse) into the boxes on the right of the screen. The category boxes will expand as questions are moved into it (example shown below).

Please read through the questions carefully. We would like you to move the 10 questions you think are most important for research to target into the "Top 10" box on the right.

As you read through the questions, please move any questions you think might be important and would like to go back over into the "questions of interest" box on the right.

You can swap and change questions from all of the boxes as often as you like. There is no right or wrong answer, just chose the questions that you think are most important for research to answer.

We are not asking you to rank the questions so you can place them in any order you like in the Top 10 group.

PLEASE make sure that 10 questions are placed in the “Top 10" box on the right. If you think there are additional questions that are important then leave them in the "questions of interest" box.

When you have selected your Top 10 questions, please click **"Finished"** in the top right corner for the next part of the survey.

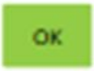

For peer review only

Questions

Are there different meaningful types of hyperacusis?
Does diet have an effect on hyperacusis?
Does exercise/physical activity have an effect on hyperacusis?
Which hearing aid settings (for managing hearing loss) are safest for people with hyperacusis?
Are online treatments effective for hyperacusis?
Are online informational resources effective for informing people about hyperacusis?
Which interventions in a school setting are useful for children with hyperacusis?
What are the most effective and acceptable strategies to raise awareness about hyperacusis?
Is private healthcare for hyperacusis more effective than NHS usual care?
Which management approach (e.g. assessment and treatment) for hyperacusis is most effective for people with learning disabilities?
What are the precautions that a dentist should use for their patients who have hyperacusis?
Which hyperacusis self-management techniques help to cope with invasive procedures (e.g. having dental work)?
Which health professionals should work together to provide the best care for adults with hyperacusis?
Is attention deficit hyperactivity disorder associated with hyperacusis?
Which strategies would make services more accessible?
What is the most effective treatment approach for hyperacusis in adults?
How should hyperacusis be assessed in different populations?
Which assessments should be conducted in the workplace to monitor and adjust for staff who have or may have hyperacusis?
What are the indicators for specialist care (e.g. psychological therapy) for someone with hyperacusis?
What is the most effective treatment approach for hyperacusis in children?
What are the early warning signs of hyperacusis?
What care is most effective for recent onset/acute hyperacusis?
Which treatment approaches are most effective for different types or severities of hyperacusis?
What is the association between hyperacusis and dementia?
Does nerve damage cause the pain associated with hyperacusis?
Is the success of hyperacusis treatment affected by the level of support provided by caregivers (e.g. parents, spouses, partners, family)?
Would restoring hearing (e.g. regenerating nerve cells) improve hyperacusis?
What is the best way of using sound in therapy for hyperacusis?
Does learned behaviour from family members with hyperacusis increase the risk of hyperacusis developing?

For peer review only

Which drugs are effective for hyperacusis?
What educational information and support is effective for hyperacusis?
Is Tinnitus Retraining Therapy effective for hyperacusis?
Is Neuromonics™ effective for hyperacusis?
Are any surgical interventions effective for hyperacusis?
What is the prevalence of hyperacusis in a general population and other specific populations (e.g. people with autism, mental health issues, learning disabilities, hearing loss)?
Is pain hyperacusis more related to hearing loss than other types of hyperacusis?
Can/should there be monitoring or screening for hyperacusis?
Can early diagnosis reduce the risk of hyperacusis getting worse?
What is the natural history (onset and changes over time) of hyperacusis?
Are hearing aids (for managing hearing loss) an effective treatment for hyperacusis?
Is there an association between hearing loss and hyperacusis?
Is peer support (e.g. support groups, mentors, families) effective for management of hyperacusis?
Does avoidance of sound improve hyperacusis or make it worse?
Is stress management effective for hyperacusis?
Is hyperacusis a symptom, a condition or a disability?
What is the level of public/employer awareness of hyperacusis?
What should be the maximum volume (e.g. in movies, concerts, headphones) to accommodate people with hyperacusis?
Can noise exposure cause hyperacusis (or make it worse)?
What preventive measures (e.g. educational information, warning labels) reduce the risk of hyperacusis developing or getting worse?
Are there certain personality traits in those prone to hyperacusis?
Which management approach (e.g. assessment and treatment) is most effective for people with hyperacusis and dementia?
What are the risk factors for developing hyperacusis or making it worse?
Is there a link between hyperacusis and non-ear-related conditions (e.g. Fibromyalgia, multiple sclerosis, diabetes, chronic fatigue, pernicious anaemia)?
Which health professionals should work together to provide the best care for children with hyperacusis?
What is the essential knowledge/training required for health professionals to appropriately refer or effectively manage hyperacusis?
Which psychological therapy (e.g. counselling, Cognitive Behavioural Therapy, mindfulness) is most effective for hyperacusis?
Is hyperacusis associated with middle ear pathology?
What is the relationship between hyperacusis and autism?
What is the relationship between mental health and hyperacusis?

For peer review only

Is hyperacusis linked to other sensitivities/conditions?
Is there an association between hyperacusis and other ear-related conditions (e.g. superior canal dehiscence syndrome, Meniere's, Waardenburg syndrome, vertigo, vestibular migraines)?
Is hyperacusis linked with trauma (e.g. brain or neck trauma, a traumatic event)?
Does stress cause or make hyperacusis worse?
Why does hyperacusis fluctuate?
What is the role of fear in hyperacusis?
Which drugs cause hyperacusis?
Is there a genetic element to hyperacusis?
What is the best work/study/healthcare environment to support people with hyperacusis?
Are any complementary (relaxation, yoga, meditation, hypnotherapy) or alternative therapies (laser therapy, vibration therapy) effective for hyperacusis?
Is hyperacusis related to physical changes in the ear or brain?
Is hyperacusis due to physical or psychological issues or is it a combination of both?
What is the best information and guidance for parents dealing with hyperacusis in their children?
What area(s) of the brain and patterns of activity is/are associated with hyperacusis?
Can otitis media with effusion cause hyperacusis?
Can infections (e.g. measles, yeast, viral) cause hyperacusis?
Can ear care (suctioning, syringing, swabbing) cause hyperacusis?
Do dental problems cause hyperacusis?
What is the best way to differentiate hyperacusis from other hearing conditions (e.g. recruitment, misophonia, Meniere's, tinnitus)?
Can hyperacusis be caused or made worse (e.g. by hormonal changes) in pregnancy?
Do musculoskeletal problems cause hyperacusis?
What are the 'red flags' for serious underlying conditions in hyperacusis?
Which criteria should be met to diagnose hyperacusis in adults/children?
Do any drugs prevent hyperacusis?
Which self-help interventions are effective for hyperacusis?
What management approach for hyperacusis is most effective for adults/children with autism?

For peer review only

Just about finished…a little bit more about you…

It is important we find out about a little about you, to make sure that we have a wide range of people with different experiences complete the survey.

What is your age or the age of your child with hyperacusis?

- ☐ under 10
- ☐ 10 - 20
- ☐ 21 - 30
- ☐ 31 - 40
- ☐ 41 - 50
- ☐ 51 - 60
- ☐ 61 - 70
- ☐ 71 - 80
- ☐ 80+

How would you describe your gender?

- ☐ Male
- ☐ Female
- ☐ I would describe it in another way

Healthcare professional only: What is your main profession?

For example, GP, ENT, Audiologist, clinical psychologist.

- ☐ Audiologist
- ☐ Audiovestibular Physician
- ☐ Chiroptactor
- ☐ Clinical Psychologist
- ☐ Clinical scientist
- ☐ ENT
- ☐ Hearing therapist
- ☐ Hynotherapist
- ☐ Paediatric audiologist
- ☐ Paediatrician
- ☐ Psychiatrist
- ☐ Nutritionist
- ☐ Other____

For peer review only

Thank you for completing this survey

Would you like to help us in the next stage of this study?

The final stage of project will be a face-to-face consensus meeting with 30 people to discuss the results from this survey and agree the Top 10 research questions. We are delighted to announce that this meeting will be held in the picturesque surroundings of the University of Nottingham (UK) on Monday 23rd July (10.00 - 17.00). **We need people with lived experience of hyperacusis, parents, carers, teachers, members of the public and healthcare professionals with experience of hyperacusis to participate in this meeting.** If you live in the UK and would like to participate, please contact us here to register for the meeting: JLAhyperacusis@nottingham.ac.uk. Places are available on a first come, first served basis. Travel for the meeting will be reimbursed and accommodation will be provided.

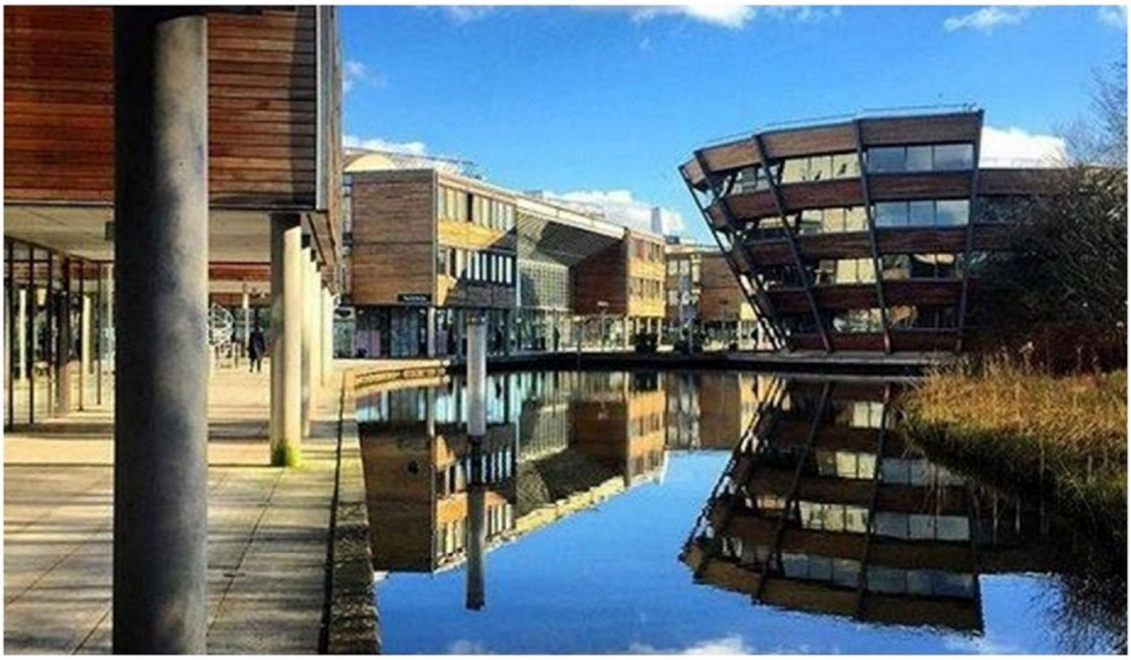

Once again thank you for your support on this project.

Please follow us on twitter for updates @HyperacusisJLA.

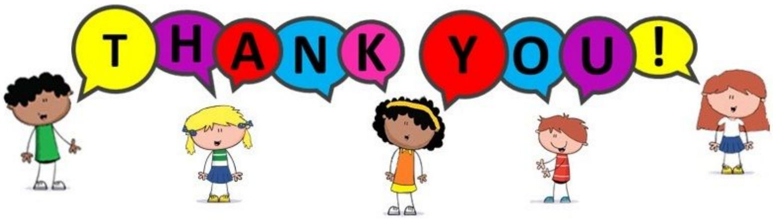

Privacy Notice

Here at the Hyperacusis James Lind Alliance Priority Setting Partnership (PSP) we take your privacy seriously. If you choose to send us your name and preferred contact details, we will only use this information for the purposes of contacting you to invite you to participate in future activities related to this PSP, which you might be interested in. There are no consequences to you if you do not want to provide your name and contact details.

The lawful basis for holding this data is that there is a legitimate interest for you to be contacted about future PSP activities. This helps the PSP ensure it listens to a variety of perspectives. The only people who have access to your name and contact details is the Administrator of this PSP and the research Lead. Your name and contact details are not linked to the survey in any way, and are not shared with anyone else. Your information is not subjected to any automatic processing or decision making.

Your name and contact details will be deleted at latest when the activities of this PSP close in August 2018. You have the right to see what information we hold about you, to correct it, and to ask us to delete it, at any time.

If you would like any further information or have any questions, please contact the PSP Administrator, Kathryn Fackrell on JLAhyperacusis@nottingham.ac.uk or look at our website: http://www.hearing.nihr.ac.uk/research/hyperacusis-PSP

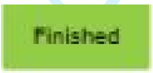

For peer review only

Supplemental appendix 4: Categories and sub-categories with example questions

Categories<br>Sub-categories		Example question
**Causes/Risk factors**		
Genetics	194.2	Are there genetic or epigenetic factor that impact the likelihood of getting hyperacusis?
Pregnancy	123.1	Can hyperacusis be caused by hormonal changes in pregnancy?
Physical or psychological	181.2	Is it triggered by worry about hearing a loud noise or can a loud noise in itself trigger the condition?
Nerve damage	130.1	Can muscular damage or wear and tear to jaw joints or neck cause hyperacusis?
Treatment	162.1	Does the insertion of cotton swabs deep inside the ear canal cause hyperacusis?
Infections	171.1	Does glue ear cause hyperacusis?
Diet/Exercise	262.3	Does diet affect hyperacusis?
Behaviour	112.2	Can hyperacusis be learned?
Exposure	163.8	Can exposure to loud sounds worsen or cause hyperacusis?
**Exacerbation**	98.3	Is there proof that overprotecting exacerbates hyperacusis?
**Classification**	243.3	Should hyperacusis be categorised differently based on its underlying causes rather than assuming they're all related?
**Prevalence**	63.3	What are the percentages of co morbidity across these 3 conditions (hyperacusis, hearing loss and tinnitus)?
**Natural history**	292.2	What is the natural progression/prognosis?
**Mechanisms**	105.11	What role, if any, does the cochlear feedback mechanism have in hyperacusis?
**Links with other conditions**		
Attention Deficit & Hyperactivity disorder	25.3	Is hyperacusis associated with other medical conditions/disabilities such as ADHD?
Autism	111.1	Is there a link between Autism and hyperacusis?
Dementia	288.1	Is hyperacusis or a type a hyperacusis a common experience in dementias?
Hearing	94.3	What is the relationship between hyperacusis and hearing loss?
Non-ear-related	297.1	Is there links between hyperacusis and chronic fatigue syndrome?
Other sensitivities	139.3	Do people with hyperacusis have other CNS symptoms ...e.g. sensitivity to bright light?
Personality	237.11	Does personality affect patients with hyperacusis?
Mental health	287.2	What role does depression and anxiety, and other mental health issues, play in how it affects people?

For peer review only

Trauma	222.3	Is it connected to head injury?
**Assessment**	39.3	What audiological test battery can be used by audiologists to detect hyperacusis and help monitor progress or regression in their patient's follow up consultation sessions?
**Management**		
Amplification	251.6	Can there be a hearing aid that has some kind of built-in equalizer so that certain frequencies can be lowered/adjusted before they reach ears?
Associated conditions	306.4	How can management of hyperacusis be reasonably adjusted for adults with intellectual disabilities?
Complementary/ alternative therapies	161.4	Does yoga improve hyperacusis?
Digital intervention	5.6	How useful /effective is online treatment for hyperacusis?
Effective treatment (general)	18.4	What is the efficacy of the current treatment strategies?
Medication	67.5	Can Selective Serotonin Reuptake Inhibitors help adults with hyperacusis?
Self-management	178.4	What can I do in my own time while I wait for the professional treatment to start, to speed up recovery?
Sound therapy	31.15	Are sound generators effective in the management of hyperacusis in children?
Surgical	147.1	How does a stapedectomy cause hyperacusis?
Tinnitus Retraining Therapy	216.4	Does TRT based treatment help hyperacusis in the long term?
Psychological therapy	39.5	Does cognitive behavioural therapy help manage underlying anxiety to further reduce hyperacusis in patients?
Dental	286.3	In what ways can we alter dental treatment to make the experience less painful for those suffering from hyperacusis?
**Prevention**	251.8	Can hyperacusis be prevented by warning labels in concerts, album covers, music apps, headphone packaging, etc.?
**Awareness**	307.5	How to roll out better education of the condition once diagnosed for families?
**Schools**	25.12	Should children with hyperacusis receive any additional support or accommodations at school?
**Service provision & evaluation**	156.8	Should there be a joint psychology/ audiology approach to managing hyperacusis?-
**Training**	221.21	Is specialty training available to otolaryngologists or audiologists regarding hyperacusis?
**Support**	33.6	Do those who experience Hyperacusis benefit from attending support groups?

For peer review only

BMJ Open

Identifying and prioritising unanswered research questions for people with hyperacusis: the James Lind Alliance Hyperacusis Priority Setting Partnership

Journal:	*BMJ Open*
Manuscript ID	bmjopen-2019-032178.R1
Article Type:	Original research
Date Submitted by the Author:	10-Sep-2019
Complete List of Authors:	Fackrell, Kathryn; University of Nottingham, NIHR Nottingham Biomedical Research Centre; University of Nottingham, Hearing Sciences, Division of Clinical Neuroscience, School of medicine<br>Stratmann, Linda; University of Nottingham, NIHR Nottingham Biomedical Research Centre<br>Kennedy, Veronica; Bolton NHS Foundation Trust, Department of Paediatric Audiology<br>MacDonald, Carol; University of Stirling, Department of Psychology<br>Hodgson, Hilary; University of Nottingham, NIHR Nottingham Biomedical Research Centre<br>Wray, Nic; University of Nottingham, NIHR Nottingham Biomedical Research Centre<br>Farrell, Carolyn; University of Nottingham, NIHR Nottingham Biomedical Research Centre<br>Meadows, Mike; University of Nottingham, NIHR Nottingham Biomedical Research Centre<br>Sheldrake, Jacqueline; The Tinnitus and Hyperacusis Centre<br>Byrom, Peter; Peter Byrom Audiology Ltd<br>Baguley, David; University of Nottingham, NIHR Nottingham Biomedical Research Centre; Nottingham University Hospitals NHS Trust, Nottingham Audiology Services<br>Kentish, Rosie; University of Nottingham, NIHR Nottingham Biomedical Research Centre<br>Chapman, Sarah; Cochrane UK<br>Marriage, Josephine; Children’s Hearing Evaluation & Amplification Resource<br>Phillips, JS; Norfolk and Norwich University Hospital NHS Trust, Department of Otolaryngology<br>Pollard, Tracey; Action on Hearing Loss<br>Henshaw, Helen; University of Nottingham, NIHR Nottingham Biomedical Research Centre; University of Nottingham, Hearing Sciences, Division of Clinical Neuroscience, School of medicine<br>Gronlund, Toto; James Lind Alliance, NIHR Evaluation, Trials and Studies Coordinating Centre<br>Hoare, Derek; University of Nottingham, NIHR Nottingham Biomedical Research Centre; University of Nottingham, Hearing Sciences, Division of Clinical Neuroscience, School of medicine
Primary Subject	Ear, nose and throat/otolaryngology

Heading:	
Secondary Subject Heading:	Evidence based practice, Research methods
Keywords:	Hyperacusis, James Lind Alliance, Research, Management, Assessment

SCHOLARONE™
Manuscripts

Identifying and prioritising unanswered research questions for people with hyperacusis: the James Lind Alliance Hyperacusis Priority Setting Partnership

Kathryn Fackrell[1,2,3], Linda Stratmann[1], Veronica Kennedy[4], Carol MacDonald[5], Hilary Hodgson[1], Nic Wray[1], Carolyn Farrell[1], Mike Meadows[1], Jacqueline Sheldrake[6], Peter Byrom[7], David M Baguley[1,2,8], Rosie Kentish[1], Sarah Chapman[9], Josephine Marriage[10], John Phillips[11], Tracey Pollard[12], Helen Henshaw[1,2], Toto Anne Gronlund[13], Derek J Hoare[1,2]

[1] National Institute for Health Research Nottingham Biomedical Research Centre, Nottingham, UK

[2] Hearing Sciences, Division of Clinical Neuroscience, School of medicine, University of Nottingham, Nottingham, UK

[3] National Institute for Health Research Evaluation, Trials and Studies Coordinating Centre, University of Southampton, Southampton, UK

[4] Department of Paediatric Audiology, Bolton NHS Foundation Trust, Bolton, UK

[5] Department of Psychology, University of Stirling, Stirling, UK

[6] The Tinnitus and Hyperacusis Centre, London, UK

[7] Peter Byrom Audiology Ltd, Sheffield, UK

[8] Nottingham Audiology Services, Nottingham University NHS Trust, Nottingham, UK

[9] Cochrane UK, Oxford, UK

[10] Children's Hearing Evaluation & Amplification Resource, London, UK

[11] Department of Otolaryngology, Norfolk and Norwich University Hospitals NHS Foundation Trust, Norwich, UK

[12] Action on Hearing Loss, London, UK

[13] James Lind Alliance, National Institute for Health Research Evaluation, Trials and Studies Coordinating Centre, Southampton, UK

Corresponding author: Kathryn Fackrell, NIHR Nottingham Biomedical Research Centre, Ropewalk House, 113 The Ropewalk, Nottingham, NG1 5DU, UK

Email: kathryn.fackrell@nottingham.ac.uk

For peer review only - http://bmjopen.bmj.com/site/about/guidelines.xhtml

ABSTRACT:

Objective

To determine research priorities in hyperacusis that key stakeholders agree are the most important.

Design/setting

A priority setting partnership using two international surveys, and a UK prioritisation workshop, adhering to the six-staged methodology outlined by the James Lind Alliance.

Participants

People with lived experience of hyperacusis, parents/carers, family and friends, educational professionals and healthcare professionals who support and/or treat adults and children who experience hyperacusis, including but not limited to surgeons, audiologists, psychologists, and hearing therapists.

Methods

The priority setting partnership was conducted from August 2017 to July 2018. An international identification survey asked respondents to submit any questions/uncertainties about hyperacusis. Uncertainties were categorised, refined, and rephrased into representative indicative questions using thematic analysis techniques. These questions were verified as “unanswered” through searches of current evidence. A second international survey asked respondents to vote for their top 10 priority questions. A shortlist of questions that represented votes from all stakeholder groups was prioritised into a top 10 at the final prioritisation workshop (UK).

Results

In the identification survey, 312 respondents submitted 2730 uncertainties. Of those uncertainties, 593 were removed as out of scope, and the remaining were refined into 85 indicative questions. None of the indicative questions had already been answered in research. The second survey collected votes from 327 respondents, which resulted in a shortlist of 28 representative questions for the final workshop. Consensus was reached on the top 10 priorities for future research, including identifying causes and underlying mechanisms, effective management, and training for healthcare professionals.

Conclusions

These priorities were identified and shaped by people with lived experience, parents/carers, and healthcare professionals, and as such are an essential resource for directing future research in hyperacusis. Researchers and funders should focus on addressing these priorities.

For peer review only - http://bmjopen.bmj.com/site/about/guidelines.xhtml

STRENGTHS AND WEAKNESSES OF THIS STUDY

1. This is the first time patients and healthcare professionals in hyperacusis have worked together to identify priorities for research
2. Used established James Lind Alliance methodology to systematically and transparently identify top research priorities
3. Survey responses from key stakeholders all over the world, which led to representative priorities for the UK, but with applicability internationally.
4. Some final indicative questions are broad and may be open to interpretation

For peer review only

For peer review only - http://bmjopen.bmj.com/site/about/guidelines.xhtml

INTRODUCTION

Hyperacusis is a hearing disorder involving an increased sensitivity or decreased tolerance to sound at levels that would not trouble most individuals. It differs from recruitment (a narrowing of the auditory dynamic range due to hearing loss), misophonia (an acquired aversive reaction to specific human generated sounds), and phonophobia (persistent, abnormal, and unwarranted fear of sound)[1–3]. For the person experiencing hyperacusis, everyday sounds of various loudness, such as the sound of the domestic appliances or electric hand dryers, can be uncomfortable or painful, and are perceived much louder and more intense than they are. This can be frightening and overwhelming and can cause anxiety and distress. Hyperacusis occurs as a primary complaint but is also commonly associated with other conditions including tinnitus, head trauma, depression, dementia, posttraumatic stress syndrome, and fibromyalgia [4]. Current estimates suggest that hyperacusis is experienced by 3.7% of children [5], and up to 9.2% of adults in a general population [6], with only 2% formally diagnosed by a physician[7]. Prevalence is particularly high in certain populations, for example, 65% of children and adults with Asperger's Syndrome or Autism Spectrum Disorder [8,9], and 95% of children and young adults with Williams syndrome experience hyperacusis [10,11].

Often chronic and disabling, hyperacusis can have a significant effect on daily life. For example, it can interfere with education, the ability to work, and/or participation in social and family life. In a recent cohort study involving 357 adult patients, 25 distinct domains of hyperacusis-associated problems were identified, including fear, avoidance behaviours, pain, activity limitations and impairments to quality of life such as reduced ability to work and socialise [12]. For children with hyperacusis, school environments (including lunch rooms and classrooms) can be challenging places, and strategies need to be in place to ensure that their wellbeing and educational needs are met [13]. People experiencing hyperacusis often become isolated and lose independence, avoiding situations where noise levels are outside their control. This not only affects the individual but also their family or carers. Many thousands of people experiencing hyperacusis or their family members turn to support and information provided online through Facebook, forums, and other social media [14].

Hyperacusis remains medically unexplained with no definitive diagnosis, aetiology, or cure [4]. For clinicians, the focus is on helping people manage their symptoms, not “cure” their hyperacusis, although no formal clinical practice guidance currently exists. The structure and provision of services for hyperacusis varies across the UK, with a variety of different medical specialties (including Ear, Nose and Throat, Audiology, Audiovestibular Medicine, Clinical Psychology) involved in the management of people experiencing hyperacusis. Clinical management in general includes non-standardised assessment using sound-based tolerance tests, and questionnaires to determine and measure the

For peer review only - http://bmjopen.bmj.com/site/about/guidelines.xhtml

specific effects hyperacusis has on an individual's life [15]. Approaches to treatment include sound therapy, counselling, and cognitive behaviour therapy (CBT) [16] but there is little or no evidence of their effectiveness. Indeed, despite its prevalence and clinical implications, hyperacusis research is in its infancy and there are many controversies in the field [17–19]. For example, only two randomised controlled trials specifically testing hyperacusis treatments have been conducted [16], and the only systematic review on hyperacusis examines prevalence in children and adolescents [20]. With such an open field, it is essential to identify and address research priorities that are immediately relevant and important to those affected by hyperacusis, and those who provide care for them. A number of different approaches exist, with some such as Child Health Nutrition Research Initiative (CHNRI) method, James Lind Alliance Method and Combined Approach Matrix, having well-defined structure [21]. In the UK, the James Lind Alliance (JLA), a non-profit initiative hosted by the National Institute of Health Research (NIHR), offers one of most established and pragmatic processes for prioritising health research questions. The JLA guiding principles are to bring together patients, carers, and healthcare professionals in Priority Setting Partnerships (PSP) to identify and agree priorities for research. The JLA provides established step-by-step methodology to manage and complete a PSP, and ensures that all processes are accountable and transparent.

The aim of this study was (i) to identify the questions about hyperacusis that are important to people with lived experience, parents/carers, family, educational professionals and healthcare practitioners involved in the care of people who have hyperacusis and (ii) to prioritise these questions according to the relevance and importance to these groups to direct future research and funding in hyperacusis.

METHODS

The hyperacusis PSP took place over an 11-month period between August 2017 and July 2018. The six-stage methodology adhered to the PSP process prescribed by the JLA (Figure 1). Documentation produced for the PSP, including the protocol, posters and initial survey are published on the JLA website (http://www.jla.nihr.ac.uk/priority-setting-partnerships/hyperacusis/).

Stage 1. Establishing a working partnership

The research leads (KF & DH) from NIHR Nottingham Biomedical Research Centre (BRC), with support from our user organisation representative (LS), initiated the PSP and appointed a steering group to oversee and contribute to the PSP process. The steering group included people with lived experience of hyperacusis, a parent of a child living with hyperacusis, members of organisations who support those with hyperacusis, and healthcare professionals (audiologists, clinical psychologists, ENT and audiovestibular medicine representatives) with experience of working with and supporting those

For peer review only

(adult and children) with hyperacusis (Supplemental appendix 1). The researchers (KF, DH, HHe) were allowed to attend the steering group meetings but did not participate in the prioritisation process. In order to reach a wide range of stakeholders, a small number of relevant partner organisations were identified through Steering group members with established links (Supplemental appendix 2). A JLA adviser acted as neutral facilitator for the PSP, providing support and guidance throughout the whole PSP process, ensuring that there were equal contributions from all key stakeholders and that at all times the PSP was conducted in a fair and transparent way. The PSP coordinator and information specialist (KF) managed the surveys and data, communication, and performed the analysis and literature searches. The steering group directed and advised on all stages of the PSP, including the scope and relevant target stakeholders (Figure 1.).

Patient and Public Involvement

Patient and public involvement is a key component of the JLA process. People with lived experience and parents/carers of those with hyperacusis were included in the steering group for the PSP. As steering group members, they were actively involved in all aspects of the project, including co-producing all materials, undertaking thematic analysis on subsets of submitted uncertainties, and reviewing all documentation. The dissemination strategy, developed with the steering group, included engagement with members of the public through plain English summaries, charity magazine articles, and an Evidently Cochrane blog co-produced with our patient representatives (https://www.evidentlycochrane.net/hyperacusis-what-need-to-know/).

*** Insert Figure 1. ***

Stage 2. Consultation and agreeing scope

The steering group raised concerns over the numerous definitions associated with hyperacusis. Definitions in the literature include but are not limited to “*decreased sound tolerance and difficulty adjusting to sudden shifts in volume*” [22], “*a heightened awareness of sound*”[23] or “*hypersensitivity to sound*”[24], “*intolerance to everyday sounds that causes significant distress and impairment in social, occupational, recreational, and other day-to- day activities*”[25] and “*negative reactions following exposure to sound that would not evoke the same response in an average listener”*[26]. Patient representatives passionately felt that the definition needed to state that it was a hearing condition, and that a description of the experience had to be provided. Therefore, using evidence from a recent scoping review[16] and the experience of steering group members, we defined hyperacusis

For peer review only - http://bmjopen.bmj.com/site/about/guidelines.xhtml

as "*a hearing disorder involving an increased sensitivity or decreased tolerance to sound at levels that would not trouble most individuals. For the person experiencing hyperacusis everyday sounds can be unpleasant, intense, frightening, painful, and overwhelming and can cause anxiety and distress*."

All uncertainties related to hyperacusis for both adults and children were considered within scope. For example, the scope included uncertainties about the (i) causes, (ii) assessment, (iii) management (treatment, rehabilitation, self-management, interventions) of hyperacusis; (iv) prevention and education, (v) care and support; and (vi) healthcare services for hyperacusis. To be inclusive and ensure a representative range of hyperacusis uncertainties were identified, the steering group made the decision that the initial survey and interim prioritisation would be available internationally. However, due to funding restrictions it was agreed that the final prioritisation workshop would involve UK-based representatives only.

Stage 3. Identification of potential research questions

An online and paper version of the survey were co-produced with the steering group. The survey consisted of an explanation of the study and what was required, six open questions relating to each element of the scope (e.g. What question(s) about the *how hyperacusis is assessed and/or diagnosed* would you like to see answered by research?) with example questions (e.g. How do clinicians check for [xxx] in children?) and an open general question (Do you have any other questions that you feel are important for researchers to answer but do not fall in the areas above?). Participants were instructed that each question was optional and that they could write as much or as little as they liked for each. Participants were also asked to complete an optional demographic information section (stakeholder group, age, gender, country of residence, ethnic background, healthcare professionals' main profession, and any other hearing conditions). Participants answered the survey anonymously and were instructed to contact the study coordinator (KF) via a study-specific email address if they wanted to be involved in the next stages of the study.

A pilot phase was used with the steering group and researchers at NIHR Nottingham BRC (including a public and patient involvement manager) to check that the survey was written clearly, understandable, and easy to complete using the online survey software (www.surveymonkey.com). The text in the survey was refined to provide clarity on the number of questions that participants had to complete (i.e. each question was optional). The survey was circulated via steering group members (including to the hyperacusisresearch.net international network based in the US), partner organisation contact lists, promotion in newsletters and national conferences, printed posters in clinics, and through dedicated social media channels (including to the international membership of the Hyperacusis Support & Research Facebook group which is led by UK and US administrators). In

For peer review only - http://bmjopen.bmj.com/site/about/guidelines.xhtml

addition to submitted uncertainties, we also searched for any research uncertainties highlighted in reviews of hyperacusis.

Stage 4. Refining research questions and identification of existing literature

The aim of this stage was to categorise and refine uncertainties, verify them as uncertainties and create a list of indicative questions for the next stage. A qualitative thematic analysis approach was taken to categorise and refine uncertainties, and generate a smaller number of representative indicative questions. This involved (i) data familiarisation (reading and re-reading submissions), (ii) developing codes for uncertainties, (iii) identifying potential categories and sub-categories, and finally (iv) reviewing and refining final categories and sub-categories. Based on the categorisation, the uncertainties were rephrased into representative indicative research questions. The analysis was undertaken by the information specialist with input from steering group members throughout. The decision about whether a submission was out of scope was initially decided by the information specialist and subsequently reviewed by the steering group. Submissions were excluded based on criteria set by the steering group before the analysis was conducted. The criteria was any uncertainties (i) outside of scope (e.g. uncertainties about misophonia or recruitment alone), (ii) advice or information requests, and (iii) broad or ambiguous submissions. Because a previous PSP on tinnitus had ranked a research priority on the relationship between tinnitus and hyperacusis in their top 10 [27], the steering group decided that any submissions related to tinnitus would also be excluded. Where more than one uncertainty was identified in the same submission, the response was duplicated and assigned to the appropriate category. The final categories and refined questions with the original submitted uncertainties were reviewed by all steering group members to ensure that the final indicative questions were understandable and true representations of the original uncertainties. The steering group agreed the final list of indicative questions.

The verification process involved a review of current evidence for each indicative question to determine whether the questions were 'true uncertainties' or had already been answered by research. Given the broad scope, a comprehensive search approach was taken to ensure that all available evidence for indicative questions was identified. The Cochrane Ear, Nose and Throat Disorders Group Trials Register; the Cochrane Central Register of Controlled Trials (CENTRAL); PubMed; Embase; International Standard Randomised Controlled Trial Number (ISRCTN) registry; ClinicalTrials.gov; Google Scholar; and Google were searched from inception of each question to May 2018 for systematic reviews, past and current trials, scoping reviews, and clinical guidelines (including guidelines produced for hearing and tinnitus to check whether there was any evidence presented for hyperacusis) relating to the indicative questions. All evidence was recorded for each indicative question with links to journal papers. Indicative questions were only considered 'true uncertainties'

For peer review only - http://bmjopen.bmj.com/site/about/guidelines.xhtml

where there was no evidence available, or was of low quality (i.e. likely further evidence would change the estimate of the effect), or where the available systematic reviews needed updating or indicated continuing uncertainties. The steering group reviewed all evidence and agreed a final list of questions which were included in the interim prioritisation survey.

Stage 5. Interim prioritisation

The purpose of this stage was to reduce the long list of questions to a shorter manageable by asking a wide range of people with lived experience of hyperacusis, and professionals to prioritise the indicative questions they considered most important. An online survey was created for this purpose using an interactive card-sorting software (www.optimalworkshop.com). The refined list of indicative questions was presented in a randomised sequence of cards for each participant on the screen. Participants were asked to read all the questions and select (drag and drop) the 10 questions that they thought were most important for research, in any order they wished (they were not asked to rank the questions). To help with the process of identifying their top 10 questions, an intermediate "questions of interest" box was included as a holding pen for questions they wished to consider. Participants were informed that they could swap and change questions from the boxes as often as they liked. Before taking part in the card sorting, participants were required to specify the stakeholder group(s) that applied to them. Once again, all survey responses were anonymous. Optional demographic information was collected (age, gender, healthcare professionals' main profession) and participants were invited to register their interest to attend the prioritisation workshop via email (Supplemental appendix 3). The survey was piloted with the steering group and researchers from NIHR Nottingham BRC to check that all instructions were clear and understandable, and to confirm the usability of the software. Instructions were refined to ensure clarity on the minimum number of questions to include in the "Top 10" box. The survey was circulated as an open invitation that was not restricted to those who had completed the first survey. The invitation was also sent to all people who had registered an interest in participating in the next stage following the initial survey, and circulated via steering group members, partner organisation contact lists, promotions in newsletters and at tinnitus support groups, printed posters in clinics, and through dedicated social media channels.

All indicative questions that were moved to the "Top 10" box were ranked based on the number of summed responses for each stakeholder group. For the analysis, weighted quantitative methods were used, in which the summed responses from professionals were weighted based on the number of responses from people with lived experience. This method was used, and agreed by the steering group, to ensure that the final indicative questions for the workshop were representative of all groups (i.e. of equal significance to votes from across all stakeholder groups). The results of the analysis were reviewed. The steering group also decided that if questions that captured under-represented groups

For peer review only - http://bmjopen.bmj.com/site/about/guidelines.xhtml

(mental health, autism, dementia, learning difficulties) were not represented in the top set then these questions would be taken forward to the final workshop and the decision was made to take a top set of approximately 25-30 indicative questions to the workshop..

Stage 6. Final prioritisation workshop

A one-day workshop with representatives from each stakeholder group was held at the University of Nottingham in July 2018. The purpose of the workshop was to establish consensus on the top 10 research priorities for hyperacusis through a blend of whole group and sub-groups discussions facilitated by independent JLA advisers with no experience of hyperacusis. The workshop was chaired by the JLA adviser for this PSP (TAG). Remote participation to the final workshop was considered by the steering group but determined not feasible due to volume-control issues for representatives living with hyperacusis and small/large group discussion format. Therefore, only UK-based representatives were invited to participate in the final workshop. Healthcare and educational professionals, people with lived experience of hyperacusis, and parents of children living with hyperacusis were invited to participate via contact details submitted following the two surveys, organisation contact lists, and social media. Participants were selected with the aim of 50:50 representation for "lived experience" (including adults, children, and parents) and professionals. A small number of steering group members were encouraged to participate in the final workshop. Attending steering group members ensured all participants were supported with information on the PSP processes and any other support as required.

Using a modified nominal group process, participants were divided into three groups with mixed representatives from people with lived experience and professionals. Each group worked together initially discussing their own personal priorities. In the second round, cards with the indicative questions printed on them were laid out for the group to view, loosely ordered to reflect the discussion in the first round. The group were tasked to work together to rank the questions from most important to least. On the back of the cards were the results from the interim prioritisation survey to assist with decision-making, if needed. The rankings from each group were combined, and the membership of the groups was changed so that each group now had someone from one of the other two groups (again with mixed representatives in each). These new small groups were all presented the combined rankings from the previous round, and tasked to review and adjust the ranking to reflect the consensus of the group. The rankings from each group were again combined and finally, the whole group came together to discuss and adjust this combined ranking, until whole group consensus was reached on the top 10 priorities for hyperacusis.

For peer review only - http://bmjopen.bmj.com/site/about/guidelines.xhtml

RESULTS

Figure 2 shows a flow diagram of the study.

*** Insert Figure 2. ***

Demographics of initial survey respondents

Two-thousand, three-hundred and seventy uncertainties about hyperacusis were submitted, online and on paper, by 312 respondents, from October 2017 to January 2018. The number of uncertainties submitted per respondent ranged from 1 to 37, with the average number submitted per respondent being 10. Respondents included people with lived experience (58%), healthcare professionals from a range of disciplines (28%), parents/carers (7%), and family and friends (1%) of those with hyperacusis. A small number of educational professionals (1%) also submitted uncertainties on the school environment and children living with hyperacusis (Table 1). Several respondents also identified themselves as having an additional role, including respondents with lived experience of hyperacusis who also identified themselves as a parent (n=1), healthcare professional (n=1) or educational professional (n=1) (Table 1). Respondents were mainly residents in the United Kingdom (65%), America (13%) and Ireland (4%), with remaining residing in various countries in Europe, Asia and Africa. Demographics of the survey respondents are presented in Table 1. No additional questions were identified in the research literature that were not already covered by the uncertainties submitted.

*** Insert Table 1. ***

Refinement of research questions and identification of existing literature

Of the 2370 submitted uncertainties, 593 were removed as out of scope. The remaining 1777 uncertainties were subjected to thematic analysis and generated 15 categories (Figure 2). Each category grouped at least two uncertainties submitted by more than one respondent from the stakeholder groups. Sub-categories were generated for the larger categories (causes, management and links with other conditions) to facilitate the process of rephrasing uncertainties into representative indicative questions (Supplemental appendix 4). The grouped uncertainties were refined, and 85 indicative questions were formulated as representative of the original uncertainties.

For peer review only - http://bmjopen.bmj.com/site/about/guidelines.xhtml

As a result of discussions, the steering group decided to leave some indicative questions as generic in terms of age (i.e. specifying children or adults). This would allow for researchers to address these from both angles and therefore increasing the number of possible priorities addressed. The existing literature did not provide any high quality evidence that addressed any of the indicative questions, therefore all 85 indicative questions were included in the interim prioritisation.

Interim priorities determined from second survey

The interim prioritisation survey opened in May 2018 and closed in July 2018. Three-hundred and twenty-seven respondents voted for their top 10 indicative research questions. Respondents were people with lived experience (67%), healthcare professionals (25%) and then a small number of parents/carers (6%), family and friends (1%), and educational professionals (1%). Demographics of the survey respondents are presented in Table 1. As expected, there was some imbalance in the priorities of different stakeholder groups. However, the weighted ranked scores for all 85 questions were reviewed by the steering group and the top 27 indicative questions were identified as representative of the votes received by all stakeholder groups. For example, 18 of the top 25 questions voted for by people with lived experience were included in the top 27. It was agreed, as per the *a priori* criteria set by the steering group, that the top 27 indicative questions, and an additional indicative question on dementia, were all priorities (Table 2).

*** Insert Table 2. ***

Top 10 priorities

The one-day workshop held in July 2018 included 21 participants, representing people with lived experience of hyperacusis (n = 6) (including two teachers), parents of children living with hyperacusis (n = 5), and healthcare professionals (n = 10) from a range of disciplines that care for and support those with hyperacusis (including audiologists, a paediatric clinical psychologist, clinical psychologists, and an audiovestibular physician). Following this workshop, consensus was reached on a top 10 research priorities for hyperacusis (Table 3). These questions cover a broad range of topics in hyperacusis including causes, mechanisms, prevalence, management and treatment, and health-care provider knowledge and training.

Publicity

The top 10 priorities were disseminated to all participants involved in the process via a contact email address or social media and shared by steering group members and partner organisations through

For peer review only - http://bmjopen.bmj.com/site/about/guidelines.xhtml

their contact lists. The top 10 priorities were unveiled at the British Academy of Audiology national conference in November 2018 [28], published in audiology and charity magazine articles [29,30] and in a published letter in a scientific journal [31]. The list of original submitted uncertainties, the 85 indicative questions, and the final list of priorities taken to the workshop have been made available on and promoted through the JLA website (http://www.jla.nihr.ac.uk/priority-setting-partnerships/hyperacusis/).

*** Insert Table 3. ***

DISCUSSION

The hyperacusis PSP has successfully brought together people with lived experience of hyperacusis and healthcare professionals in hyperacusis to identify and agree priorities for research. These priorities were therefore identified and shaped by people with lived experience, parents/carers and healthcare professionals. Although this process did not identify radically different questions, it did highlight the sheer number of unanswered questions about hyperacusis. The top 10 priorities reflect the lack of evidence and guidance on the assessment and management of hyperacusis, including the need for training in hyperacusis. These priorities highlight the need for research to focus on the underlying mechanisms and types of hyperacusis, and prevalence of hyperacusis in specific populations. The list of priorities identified here will therefore be of interest to researchers and funders of hearing/ health research, and will hopefully direct future research and funding calls.

There are many notable strengths to this study. Firstly, people with lived experience and parents/carers were involved throughout the process, from the initial planning for the JLA with our user organisation representative, planning and overseeing the project as members of the steering group, and participation in both surveys worldwide, and in the final workshop. All of this enabled people with lived experience and parents/carers to have the opportunity for a voice and to influence the future priorities for research on hyperacusis. Secondly, this PSP also took an inclusive approach to gathering the uncertainties and interim voting by having two international online surveys, and although the final consensus on the priorities was reached with UK representatives only, the top 10 priorities, and the longer list of priorities, have international relevance as they represent unanswered questions submitted by people with lived experience, parents/carers and healthcare professionals from around the world. However, finer grain analysis of the initial submissions by country was not meaningful because there were only a small number of submissions from most countries.

For peer review only - http://bmjopen.bmj.com/site/about/guidelines.xhtml

Predominantly responses were received from participants in the UK and US, which is partly a product of the promotional routes taken in the online surveys. However, one notable difference was observed, that the initial submissions by US participants frequently asked about reimbursement of healthcare costs, whereas for the UK this is not an issue. Therefore, whilst it is possible that some health service provision and evaluation questions are less relevant in some countries, the remaining indicative questions collected in this PSP should be considered unanswered priorities for all. Thirdly, there were notable differences in the interim prioritisation between people with lived experience and healthcare professionals. For example, healthcare professionals were more likely to prioritise questions relating to effective treatments for both adults and children and differentiating hyperacusis, whereas people with lived experience were more likely to prioritise causes then effective treatments and differentiating hyperacusis (Table 2). However, by using weighted ranking, the top 10 reflected the mixed priorities from all stakeholder groups, of which five were in the top 10 for people with lived experience and parents/carers during the interim prioritisation and six were in the top 10 for professionals during the interim prioritisation. Finally, by using the JLA process for prioritisation, independent JLA advisors supported and guided the whole PSP process, including managing any potential conflicts arising from differences in perspectives and power relations between healthcare professionals and people with lived experience of hyperacusis and ensuring that all stakeholders had an equal voice.

There were some challenges with the PSP process. One challenge was interpreting the submissions that were more narrative stories than specific research questions. Due to the nature of the PSP, many submissions were asking more than one question within a narrative story, especially submissions from people experiencing hyperacusis who until this point had not been asked their point of view. To ensure that all vital information and possible questions from the narratives were identified and recorded, the information specialist worked closely with steering group members to interpret the narratives. Another challenge arose in planning the JLA final face-to-face workshop; people who experience hyperacusis find that travelling and participating in large and small group discussions can lead to pain, discomfort, or distress. In order to overcome any foreseeable problems with recruiting people with lived experience, different options were discussed by the steering group. Remote participation was suggested as a potential avenue for people to take part in the workshop. However, it was decided from experience of remote steering group meetings, and advice sought from the JLA, that online discussion facilities such as Skype or teleconferences would not overcome the problems with discomfort, would be physically and mentally taxing to participants, and could impede the discussion process. The venue for the workshop was discussed. It was decided that the workshop would take place at the University of Nottingham during the summer period when there are no students on

For peer review only - http://bmjopen.bmj.com/site/about/guidelines.xhtml

campus as this would reduce the number of potential problems with loud noises. The facilities used for the workshop were quiet rooms with limited external sounds. Participants were given the option to travel the day before the workshop to allow time to recover from travelling. All facilitators and participants were aware of potential problems that could occur and a dedicated quiet room was available for anyone to go to for a break. The lead researchers were on hand to offer any additional support to participants. Therefore, although a relatively small number of people with lived experience participated in the final workshop (six people with lived experience and five parents of children with hyperacusis), it did not impede the discussions or consensus as there was balanced representation from each stakeholder group. Also, the results from interim prioritisation, where a large number people experiencing hyperacusis voted, informed the discussions.

Many of the priorities highlighted by this PSP clearly mark areas that are less well researched in hyperacusis. For example, in 2014, a two-part review of hyperacusis research [17,18] highlighted key areas in hyperacusis that were seen as important to progress, including but not limited to, properly defining and subtyping hyperacusis, identifying and understanding mechanisms and the role of the brain, and evaluating current treatments (particularly sound therapy and drugs). These topics also appeared in reviews published in 2017 [16] and 2018 [19]. These more recent reviews also reported a need for up-to-date prevalence figures and valid measurement tools. All these topics reflect the 28 priority questions identified in our interim prioritisation survey. This would suggest that the majority of recently published research in hyperacusis has not necessarily been addressing the areas of concern. A search of Google Scholar revealed that the most recent publications for hyperacusis have focused on basic research questions around causes of hyperacusis [32–34], or on research investigating hyperacusis and the workplace [35]. The only relevant ongoing trial is investigating "Exposure Therapy for Auditory Sensitivity in Autism" (ClinicalTrials.gov Identifier: NCT03206996). Although this research is important, it is somewhat disheartening to observe that the majority of the areas highlighted by a review in 2014 still remain an issue now. This prioritisation exercise highlighted the need for research to have a much greater focus on clinical needs (management approaches) and effective treatments, as well as aspects of underlying mechanisms and subtyping. With these questions now in place it is a responsibility to people with lived experience and healthcare professionals that researchers in the field pursue these questions as their priorities, and thereby minimise research waste in this field [36]. All sources of data related to these questions, whether through clinical audit, retrospective analyses, undergraduate or postgraduate projects, or bespoke grant applications, will usefully start to address these questions and build capacity in this neglected field. It will be important that researchers engage with funders to formulate feasible projects they are likely to endorse.

For peer review only - http://bmjopen.bmj.com/site/about/guidelines.xhtml

This PSP highlighted a potential challenge within current healthcare for hyperacusis. A larger number of patients engaged in each of the surveys than healthcare professionals. This could be a reflection of our current healthcare system, in which only a small number of healthcare professionals have experience in such a specialised subject as hyperacusis. Moreover, it was apparent from the list of priorities that identifying the essential training needs of healthcare professionals for each clinical speciality involved with hyperacusis is a priority. Furthermore, the number of out of scope uncertainties asking for information on hyperacusis and care and support further highlighted this problem and a clear need for greater awareness of hyperacusis. Current knowledge and awareness of hyperacusis in the UK is sadly lacking. Therefore, informational resources are needed to raise awareness of hyperacusis and provide guidance on seeking care and support. However, in order to provide this information, first service evaluations need to be conducted to provide a clear picture of the current position of hyperacusis care and identify the areas of need.

Conclusion

Hyperacusis is a topic of growing interest. Here we have identified a set of priority questions that reflect what people with lived experience, parents/carers, and healthcare professionals, want answered by research. As such researchers and funders should focus on addressing these priorities.

For peer review only - http://bmjopen.bmj.com/site/about/guidelines.xhtml

ACKNOWLEDGEMENTS

The authors wish to acknowledge the people with lived experience of hyperacusis, parents of children living with hyperacusis, families and friends, carers and educational and healthcare professionals who submitted responses to the international surveys. In addition, they would like to thank the partner organisations (Supplemental appendix 2) who supported and promoted this work, Sandra Smith for checking the literature searches and providing support at the final prioritisation workshop, the JLA for support and guidance throughout the process and all attendees and JLA facilitators (Katherine Cowan, Sheela Upadhyaya, and Toto Anne Gronlund) at the final workshop who worked tirelessly to achieve a consensus on the top 10 research questions.

FUNDING

This work was supported through grants from the British Society of Audiology and Action on Hearing Loss. The Partnership was supported by the National Institute for Health Research (NIHR) Nottingham Biomedical Research Centre (BRC).

DISCLAIMER

KF is funded by the National Institute for Health Research (NIHR Post-Doctoral Fellowship, Dr Kathryn Fackrell, PDF-2018-11-ST2-003). HHe is funded by the National Institute for Health Research (NIHR Career Development Fellowship, Dr Helen Henshaw, CDF-2018-11-st2-016). DJH is funded by the National Institute for Health Research (NIHR) Biomedical Research Centre programme. However the views expressed in this publication are those of the author(s) and not necessarily those of the NHS, the National Institute for Health Research or the Department of Health and Social Care.

COMPETING INTERESTS

DJH is vice-chair of the British Society of Audiology. KF is a member of the British Society of Audiology Tinnitus and Hyperacusis special interest group. DMB is president of the British Tinnitus Association. NW is an employee of the British Tinnitus Association.

CONTRIBUTIONS

KF and DH made the initial application to the JLA for a hyperacusis PSP. KF organised steering group meetings and teleconferences, contacted and enrolled all partner organisations, designed the surveys,

For peer review only

For peer review only - http://bmjopen.bmj.com/site/about/guidelines.xhtml

liaised with organisations throughout the PSP, promoted the PSP through a dedicated Twitter account, conducted thematic analysis and literature reviews, organised and enrolled participants for the final workshop, and drafted the final manuscript. DH organised venues and facilities for meetings and final workshop, drafted and circulated minutes of every meeting and teleconference, conducted initial thematic analysis and analysis on the interim prioritisation survey data. TAG was the JLA independent facilitator for the PSP. TAG chaired all steering group meetings and the final workshop, provided support and guidance throughout the whole PSP process to members of the steering group and the research team including reviewing documentation. LS, VK, CM, HH, NW, CF, MM, JS, PB, DMB, RK, SC, JM, JP, TP, HH attended steering group meetings, made decisions on the scope, documentation and analysis to identify indicative questions for survey and final workshop. LS, VK, CM, HH, NW, CF, MM, JS, PB, DMB, RK, SC, JM, JP, TP, HH co-developed the information presented in the surveys and promotions, commented and rephrased the 85 indicative questions and analysed and identified the 28 indicative questions for the final workshop. LS, VK, CM, CF, HH, JM, DMB, RK, JS conducted thematic analysis on a subset of submitted uncertainties. SC provided advice on promotional routes and promoted the PSP through Evidently Cochrane blog. All authors read and approved the final manuscript for submission.

For peer review only

PATIENT CONSENT

Not required.

ETHICS APPROVAL

This work did not require ethics approval as per the JLA guidance and guidance published by the NHS National Patient Safety Agency National Research Ethics Service.

PROVENANCE AND PEER REVIEW

Not commissioned; externally peer reviewed.

DATA SHARING STATEMENT

Supplementary data including all submitted original research uncertainties can be found on the JLA website at http://www.jla.nihr.ac.uk/priority-setting-partnerships/hyperacusis/

For peer review only - http://bmjopen.bmj.com/site/about/guidelines.xhtml

REFERENCES

1 McFerran D. Misophonia and Phonophobia. In: Baguley DM, Fagelson M, eds. *Tinnitus: clinical and research perspectives.* San Diego: : Plural Publishing Inc 2016. 245–60.

2 Kumar S, Tansley-Hancock O, Sedley W, *et al.* The Brain Basis for Misophonia. *Curr Biol* 2017;27:527–33.

3 Moore B. *Cochlear Hearing Loss: Physiological, Psychological and Technical Issues.* 2nd ed. Chichester, UK: : Wiley 2007.

4 Baguley DM. Hyperacusis. *J R Soc Med* 2003;96:582–5.

5 Hall AJ, Humphriss R, Baguley DM, *et al.* Prevalence and risk factors for reduced sound tolerance (hyperacusis) in children. *Int J Audiol* 2016;55:135–41.

6 Andersson G, Lindvall N, Hursti T, *et al.* Hypersensitivity to sound (hyperacusis): a prevalence study conducted via the Internet and post. *Int J Audiol* 2002;41:545–54.

7 Paulin J, Andersson L, Nordin S. Characteristics of hyperacusis in the general population. *Noise Heal* 2016;18:178–84.

8 Danesh AA, Lang D, Kaf W, *et al.* Tinnitus and hyperacusis in autism spectrum disorders with emphasis on high functioning individuals diagnosed with Asperger's Syndrome. *Int J Pediatr Otorhinolaryngol* 2015;79:1683–8.

9 Khalfa S, Bruneau N, Rogé B, *et al.* Increased perception of loudness in autism. *Hear Res* 2004;198:87–92.

10 Baguley DM, McFerran D. Hyperacusis and Disorders of Loudness Perception. In: Moller A, Langguth B, DeRidder D, *et al.*, eds. *Textbook of Tinnitus*. New York: : Springer 2011. 13–24.

11 Klein B, Mervis C. Contrasting patterns of cognitive abilities of 9-and 10-year-olds with William's syndrome or Down syndrome. *Dev Neuropsychol* 1999;16:177–96.

12 Fackrell K, Sereda M, Sheldrake J, *et al.* Why is Hyperacusis a Problem? A Qualitative Analysis of Problems Reported by Patients and Research Volunteers. In: *British Academy of Audiology's 14th Annual Conference*. Bournemouth, UK: 2017.

13 Myne S, Kennedy V. Hyperacusis in children: A clinical profile. *Int J Pediatr Otorhinolaryngol* 2018;107:80–5.

14 Fackrell K, Sereda M, Sheldrake J, *et al.* Untangling the relationship between tinnitus and hyperacusis: What are the problems reported by patients with a primary complaint of hyperacusis. In: *International Tinnitus Research Initiative Conference*. Regensburg, Germany: 2018.

15 Fackrell K, Hoare DJ. Scales and questionnaires for decreased sound tolerance. In: Baguley DM, Fagelson M, eds. *Hyperacusis and Disorders of Sound Intolerance: Clinical and Research Perspectives.* San Diego, CA: : Plural Publishing Inc 2018.

16 Fackrell K, Potgieter I, Shekhawat GS, *et al.* Clinical Interventions for Hyperacusis in Adults : A Scoping Review to Assess the Current Position and Determine Priorities for Research. *Biomed Res Int* 2017;2017.

17 Tyler RS, Pienkowski M, Roncancio ER, *et al.* A Review of Hyperacusis and Future Directions: Part I. Definitions and Manifestations. *Am J Audiol* 2014;23:402–419.

For peer review only

For peer review only - http://bmjopen.bmj.com/site/about/guidelines.xhtml

18 Pienkowski M, Tyler RS, Roncancio ER, *et al.* A Review of Hyperacusis and Future Directions: Part II. Measurement, Mechanisms, and Treatment. *Am J Audiol* 2014;23:420–436.

19 Baguley D, Hoare D. Hyperacusis: major research questions. *HNO* 2018;66:358–63.

20 Rosing SN, Schmidt JH, Wedderkopp N, *et al.* Prevalence of tinnitus and hyperacusis in children and adolescents: a systematic review. *BMJ Open* 2016;6:e010596.

21 Yoshida S. Approaches, tools and methods used for setting priorities in health research in the 21(st) century. *J Glob Health* 2016;6:010507.

22 Attri D, Nagarkar AN. Resolution of hyperacusis associated with depression, following lithium administration and directive counselling. *J Laryngol Otol* 2010;124:919–21.

23 Phillips D, Carr M. Disturbances of loudness perception. *J Am Acad Audiol* 1998;9:371–9.

24 Suchova L. Tinnitus retraining therapy--the experiences in Slovakia. *Bratisl Lek Listy* 2005;106:79–82.

25 Aazh H, Moore BCJ, Lammaing K, *et al.* Tinnitus and hyperacusis therapy in a UK National Health Service audiology department: Patients' evaluations of the effectiveness of treatments. *Int J Audiol* 2016;2027:1–9.

26 Jastreboff PJ, Jastreboff MM. Treatments for decreased sound tolerance (hyperacusis and misophonia). *Semin Hear* 2014;35:105–20.

27 Hall DA, Mohamad N, Firkins L, *et al.* Identifying and prioritizing unmet research questions for people with tinnitus: the James Lind Alliance Tinnitus Priority Setting Partnership. *Clin Investig (Lond)* 2013;3:21–8.

28 Fackrell K on behalf of the hyperacusis P steering group. Prioritising hyperacusis: The Top 10 research priorities. In: *British Academy of Audiology's 15th Annual Conference*. Liverpool, UK: 2018.

29 Hoare D, Fackrell K. Collaborating with patients on research priorities in hyperacusis: the James Lind Alliance project. *ENT News* 2018.

30 Fackrell K on behalf of the hyperacusis P steering group. Prioritising hyperacusis. *Quiet, Br Tinnitus Assoc* 2019.

31 Fackrell K, Stratmann L, Gronlund T, *et al.* Top ten hyperacusis research priorities in the UK. *Lancet* 2019;393:404–5.

32 Kompis M, Hohl A, Seifert E, *et al.* Voluntary increase of acoustic middle ear impedances with simultaneous sound attenuation associated with mild hyperacusis (VIMH). *Acta Otolaryngol* 2019;:1–6.

33 Farahani S, Nasirinezhad F, Danyali S, *et al.* Does 5, 7-Dihydroxytryptamine injection into nucleus accumbens cause hyperacusis? *Neurosci Lett* Published Online First: 7 April 2019.

34 Silverstein H, Smith J, Kellermeyer B. Stapes hypermobility as a possible cause of hyperacusis. *Am J Otolaryngol* 2019;40:247–52.

35 Paulin J, Nordin M, Nyback M-H, *et al.* Associations between hyperacusis and psychosocial work factors in the general population. *Int Arch Occup Environ Health* 2019;92:59–65.

36 Chalmers I, Glasziou P. Avoidable waste in the production and reporting of research evidence. *Lancet* 2009;374:86–9.

For peer review only - http://bmjopen.bmj.com/site/about/guidelines.xhtml

TABLES

Table 1. Demographics of respondents to the identification survey and interim prioritisation survey

Characteristics	Identification	Interim prioritisation
	n	n
**Role**		
Person with hyperacusis	181	218
Tinnitus	74	—
Hearing loss	36	—
Tinnitus and hearing loss	66	—
Parent of a child with hyperacusis	19 (2)	14 (4)
Carer of someone with hyperacusis	2 (1)	4 (5)
Friend or family member of someone with hyperacusis	3	4 (7)
Education professionals (e.g school teacher, lecturer)	3 (3)	2 (7)
Healthcare professional	87 (1)	82 (11)
Audiologist	43	38
Paediatric audiologist	7	2
ENT	2	6
Audiovestibular Physician	11	7
Clinical scientist	3	5
Hearing therapist	9	3
Speech-language pathologist	—	2
Speech and Language Therapist	—	1
Clinical Psychologist	4	
Psychiatrist	1	1
Clinical Hypnotherapist	1	—
Intensive care physician	—	1
GP	—	1
Paediatrician	1	2
Nurse	—	3
Dentist	—	1
Chiropractor and nutritionist	1	—
More than one role identified	7	34
Other	8	3
Not specified	9	—
**Age**		
Under 10	0	4
10 - 20	7	11
21 - 30	19	30
31 - 40	40	49
41 - 50	67	37
51 - 60	85	59
61 - 70	57	42
71 - 80	16	19
80+	1	2

For peer review only

Not specified	20	74
**Sex**		
Female	194	95
Male	103	152
I would describe it in another way	—	2
Not specified	5	78
**Ethnicity**		
White	276	—
Asian	8	—
Mixed/ multiple ethnic group	4	—
Arab	1	—
Black	1	—
Prefer not to say	20	—
Other (not specified)	5	—
**Country of residence**		
United Kingdom (UK)	203	—
United States of America (USA)	42	—
Ireland	11	—
Australia	6	—
Canada	6	—
New Zealand	4	—
Sweden	4	—
Netherlands	3	—
Germany	2	—
South Africa	2	—
Austria	1	—
Belgium	1	—
Brazil	1	—
Denmark	1	—
Finland	1	—
France	1	—
Greece	1	—
Portugal	1	—
Philippines	1	—
Saudi Arabia	1	—
Switzerland	1	—

For peer review only

619 **Table 2. The top 28 ranked questions from interim prioritisation survey**

		Summed responses for each stakeholder group		
**Rank**	**Research Question**	**Person with hyperacusis**	**Parents/ Carers/ family**	**Prof~**
1	What is the most effective treatment approach for hyperacusis in adults?	60	6	80
2	Which treatment approaches are most effective for different types or severities of hyperacusis?	51	6	77
3	Does avoidance of sound improve hyperacusis or make it worse?	60	8	51
4	Is hyperacusis related to physical changes in the ear or brain?	69	3	46
5	Is hyperacusis due to physical or psychological issues or is it a combination of both?	41	7	65
6	Which psychological therapy (e.g. counselling, Cognitive Behavioural Therapy, mindfulness) is most effective for hyperacusis?	18	7	85
7	What are the 'red flags' for serious underlying conditions in hyperacusis?	28	7	68
8	Which criteria should be met to diagnose hyperacusis in adults/children?	25	5	71
9	What area(s) of the brain and patterns of activity is/are associated with hyperacusis?	51	4	46
10	What is the most effective treatment approach for hyperacusis in children?	10	7	83
11	What are the risk factors for developing hyperacusis or making it worse?	37	3	54
12	What is the best way of using sound in therapy for hyperacusis?	20	2	71
13	What is the essential knowledge/training required for health professionals to appropriately refer or effectively manage hyperacusis?	27	5	57
14	What management approach for hyperacusis is most effective for adults/children with autism?	8	5	74
15	Is there an association between hyperacusis and other ear-related conditions (e.g. superior canal dehiscence syndrome, Meniere's, Waardenburg syndrome, vertigo, vestibular migraines)?	36	3	43
16	What is the best way to differentiate hyperacusis from other hearing conditions (e.g. recruitment, misophonia, Meniere's, tinnitus)?	30	3	48
17	Which self-help interventions are effective for hyperacusis?	40	7	34
18	Is hyperacusis linked to other sensitivities/conditions?	34	11	34

For peer review only - http://bmjopen.bmj.com/site/about/guidelines.xhtml

19	Does nerve damage cause the pain associated with hyperacusis?	60	5	11
20	What is the prevalence of hyperacusis in a general population and other specific populations (e.g. people with autism, mental health issues, learning disabilities, hearing loss)?	17	3	54
21	Which drugs are effective for hyperacusis?	45	3	26
22	Can noise exposure cause hyperacusis (or make it worse)?	59	6	9
23	Which interventions in a school setting are useful for children with hyperacusis?	11	7	54
24	Would restoring hearing (e.g. regenerating nerve cells) improve hyperacusis?	56	5	6
25	Are there different meaningful types of hyperacusis?	23	3	40
26	What is the relationship between mental health and hyperacusis?	20	6	40
27	What care is most effective for recent onset/acute hyperacusis?	30	1	34
28	What is the association between hyperacusis and dementia?*	16	0	9

Prof = healthcare and educational professionals. ~weighted professionals score. *Additional question on dementia with lower ranking included at final workshop as per *a priori* criteria set by steering group.

For peer review only

Table 3. Hyperacusis Priority Setting Partnership top ten priorities for future research

Priority order	Research Question
1	What is the most effective treatment approach for hyperacusis in children?
2	What is the prevalence of hyperacusis in a general population and other specific populations (e.g. people with autism, mental health issues, learning disabilities, hearing loss)?
3	Are there different meaningful types of hyperacusis?
4	What is the essential knowledge/training required for health professionals to appropriately refer or effectively manage hyperacusis?
5	Which treatment approaches are most effective for different types or severities of hyperacusis?
6	Is hyperacusis due to physical or psychological issues or is it a combination of both?
7	Which psychological therapy (e.g. counselling, Cognitive Behavioural Therapy, mindfulness) is most effective for hyperacusis?
8	What management approach for hyperacusis is most effective for adults/children with autism?
9	What is the best way of using sound in therapy for hyperacusis?
10	Which self-help interventions are effective for hyperacusis?

For peer review only

FIGURE LEGENDS

Figure 1. The six-stage methodological process for the Hyperacusis Priority Setting Partnership

Figure 2. Flow diagram of prioritisation process from submitted research questions to final prioritisation. * One additional question on dementia with lower ranking was included at final workshop as per *a priori* criteria set by steering group.

SUPPLEMENTAL MATERIAL

Supplemental appendix 1. Steering Group of the Hyperacusis Priority Setting Partnership

Supplemental appendix 2: Partner organisations

Supplemental appendix 3: Interim prioritisation survey

Supplemental appendix 4: Categories and sub-categories with example questions

For peer review only

For peer review only - http://bmjopen.bmj.com/site/about/guidelines.xhtml

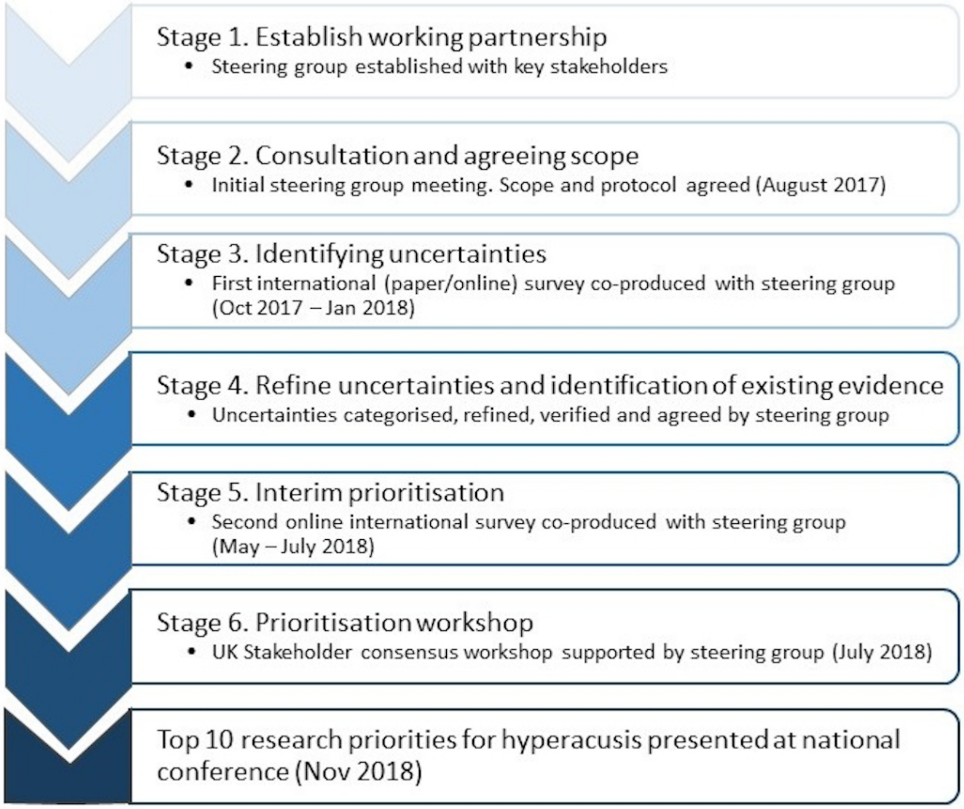

Figure 1. The six-stage methodological process for the Hyperacusis Priority Setting Partnership

90x75mm (300 x 300 DPI)

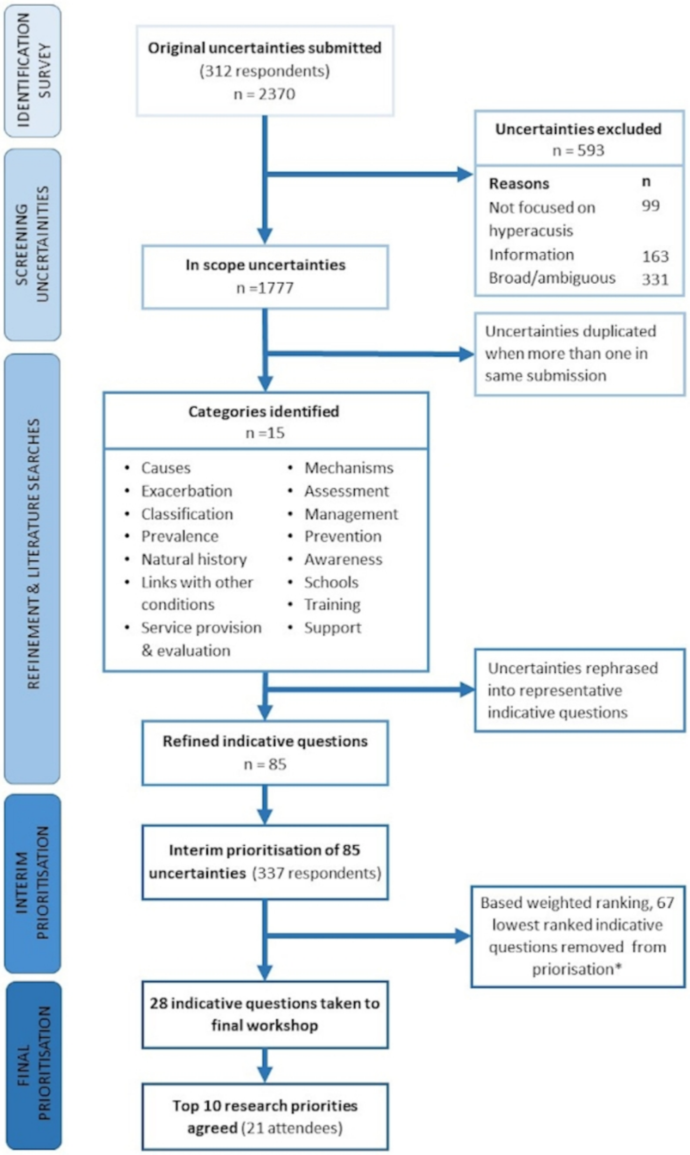

Figure 2. Flow diagram of prioritisation process from submitted research questions to final prioritisation. *One additional question on dementia with lower ranking was included at final workshop as per a priori criteria set by steering group.

90x132mm (300 x 300 DPI)

SUPPLEMENTAL MATERIALS

Supplemental appendix 1. Steering Group of the Hyperacusis Priority Setting Partnership.

Steering group member	Role
**James Lind Alliance support and guidance**	
Toto Anne Gronlund	Independent chair, representing James Lind Alliance
Katherine Cowan	Final workshop Facilitator, The James Lind Alliance
Sheela Upadhyaya	Final workshop Facilitator, The James Lind Alliance
**Non-voting members**	
Kathryn Fackrell	Study coordinator and Information specialist, Researcher, University of Nottingham
Derek Hoare	Researcher, University of Nottingham
Helen Henshaw	Researcher, University of Nottingham
**Patient representatives**	
Linda Stratmann	User organisation, founder and co-moderator of Facebook group Hyperacusis Support & Research, and person with lived experience
Tracey Pollard	Action on Hearing Loss patient representative
Nic Wray	Person with lived experience
Carolyn Farrell	Person with lived experience
Mike Meadows	Person with lived experience
Hilary Hodgson	Parent
Sarah Chapman	Cochrane UK Knowledge Broker
**Clinical representatives**	
Carol MacDonald	CBT psychotherapist and Clinical Psychology Lecturer
David Baguley	Clinical Scientist (audiology)
Jacqueline Sheldrake	Audiologist
John Phillips	ENT surgeon
Josephine Marriage	Paediatric audiologist
Peter Byrom	Audiologist
Rosie Kentish	Clinical Psychologist
Veronica Kennedy	Audiovestibular Physician

For peer review only

Supplemental appendix 2: Partner organisations

Charity organisations

Action on Hearing Loss

Space 4 Autism

Professional organisations

British Society of Audiology

British Association of Audiovestibular Physicians

National Institute of Health Research Nottingham Biomedical Research Centre

Social media outlets

Facebook group Hyperacusis Support & Research

National Institute of Health Research Nottingham Biomedical Research Centre Facebook

Study-dedicated twitter account (@HyperacusisJLA)

For peer review only

Supplemental appendix 3: Interim prioritisation survey

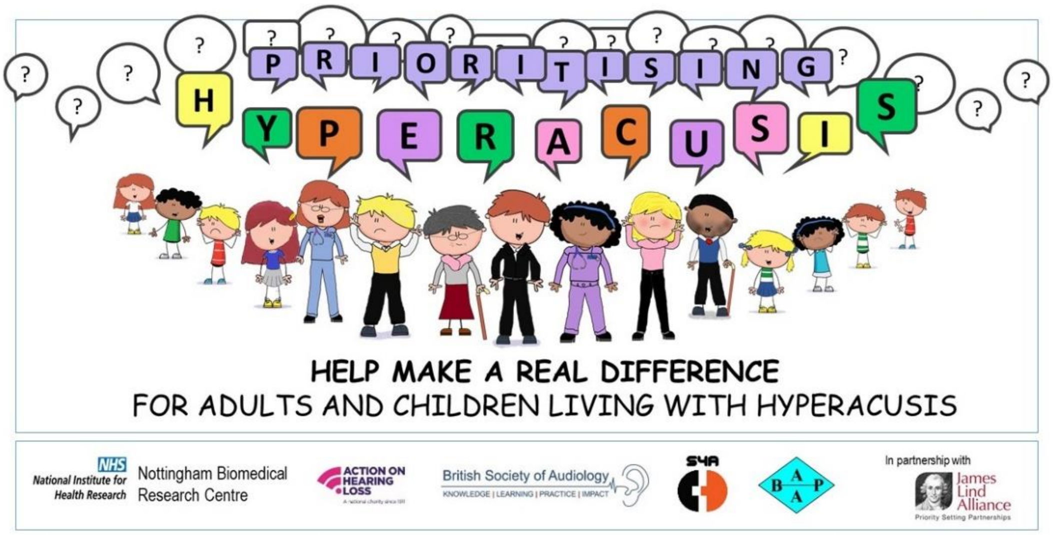

Welcome to this Hyperacusis James Lind Alliance Priority Setting Partnership survey.

Thank you for agreeing to participate!

What is hyperacusis?

Hyperacusis is a hearing disorder involving an increased sensitivity or decreased tolerance to everyday sound at levels that would not trouble most individuals. For the person experiencing hyperacusis everyday sounds can be unpleasant, intense, frightening, painful, and overwhelming and can cause anxiety affecting their quality of life.

We need you to vote for your Top 10 research questions about hyperacusis

We want to identify the Top 10 questions that research should target. Our first survey asked for people with lived experience of hyperacusis, parents, carers, teachers, members of the public and healthcare professionals who work with and support those who experience hyperacusis to submit any questions they had about hyperacusis.

In this survey you have the opportunity to choose 10 "unanswered" questions about hyperacusis that you think are the most important for research to target. Anyone with experience of hyperacusis (except researchers) can complete this survey. You do not have to have taken part in any previous studies.

The survey should not take longer than 15 minutes to complete.

Why do this?

Your responses will help us and other organisations to target research and funding for hyperacusis. This really is a unique opportunity to have your say and make sure that research addresses questions that matter to you and others like you.

Find out how on the next page...

For peer review only

Continue

PRIORITISING HYPERACUSIS

Thank you for agreeing to complete this survey. Before we begin…

We just need to know your experience with hyperacusis to make sure that we understand the voting patterns by a wide range of people with different experiences. Anyone with experience of hyperacusis can complete this survey!

Which of the following best describes you?

Please select all the groups that apply to you. For example, if you are a healthcare professional and a parent of a child with hyperacusis then select both.

- ☐ Person with hyperacusis currently or in past
- ☐ Carer of someone with hyperacusis
- ☐ Parent of a child with hyperacusis
- ☐ Family or friend of someone with hyperacusis
- ☐ Educational professional, e.g. school teacher, lecturer
- ☐ Healthcare professional
- ☐ Other

Continue

For peer review only

Instructions

On the left of the screen in a list of "unanswered" research questions about hyperacusis.

On the right of screen are two category boxes ("questions of interest" and "Top 10"). The questions on the left of the screen are designed for you to move them (drag and drop them using mouse) into the boxes on the right of the screen. The category boxes will expand as questions are moved into it (example shown below).

Please read through the questions carefully. We would like you to move the 10 questions you think are most important for research to target into the "Top 10" box on the right.

As you read through the questions, please move any questions you think might be important and would like to go back over into the "questions of interest" box on the right.

You can swap and change questions from all of the boxes as often as you like. There is no right or wrong answer, just chose the questions that you think are most important for research to answer.

We are not asking you to rank the questions so you can place them in any order you like in the Top 10 group.

PLEASE make sure that 10 questions are placed in the “Top 10" box on the right. If you think there are additional questions that are important then leave them in the "questions of interest" box.

When you have selected your Top 10 questions, please click **"Finished"** in the top right corner for the next part of the survey.

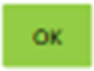

For peer review only

Questions

Are there different meaningful types of hyperacusis?
Does diet have an effect on hyperacusis?
Does exercise/physical activity have an effect on hyperacusis?
Which hearing aid settings (for managing hearing loss) are safest for people with hyperacusis?
Are online treatments effective for hyperacusis?
Are online informational resources effective for informing people about hyperacusis?
Which interventions in a school setting are useful for children with hyperacusis?
What are the most effective and acceptable strategies to raise awareness about hyperacusis?
Is private healthcare for hyperacusis more effective than NHS usual care?
Which management approach (e.g. assessment and treatment) for hyperacusis is most effective for people with learning disabilities?
What are the precautions that a dentist should use for their patients who have hyperacusis?
Which hyperacusis self-management techniques help to cope with invasive procedures (e.g. having dental work)?
Which health professionals should work together to provide the best care for adults with hyperacusis?
Is attention deficit hyperactivity disorder associated with hyperacusis?
Which strategies would make services more accessible?
What is the most effective treatment approach for hyperacusis in adults?
How should hyperacusis be assessed in different populations?
Which assessments should be conducted in the workplace to monitor and adjust for staff who have or may have hyperacusis?
What are the indicators for specialist care (e.g. psychological therapy) for someone with hyperacusis?
What is the most effective treatment approach for hyperacusis in children?
What are the early warning signs of hyperacusis?
What care is most effective for recent onset/acute hyperacusis?
Which treatment approaches are most effective for different types or severities of hyperacusis?
What is the association between hyperacusis and dementia?
Does nerve damage cause the pain associated with hyperacusis?
Is the success of hyperacusis treatment affected by the level of support provided by caregivers (e.g. parents, spouses, partners, family)?
Would restoring hearing (e.g. regenerating nerve cells) improve hyperacusis?
What is the best way of using sound in therapy for hyperacusis?
Does learned behaviour from family members with hyperacusis increase the risk of hyperacusis developing?

For peer review only

Which drugs are effective for hyperacusis?
What educational information and support is effective for hyperacusis?
Is Tinnitus Retraining Therapy effective for hyperacusis?
Is Neuromonics™ effective for hyperacusis?
Are any surgical interventions effective for hyperacusis?
What is the prevalence of hyperacusis in a general population and other specific populations (e.g. people with autism, mental health issues, learning disabilities, hearing loss)?
Is pain hyperacusis more related to hearing loss than other types of hyperacusis?
Can/should there be monitoring or screening for hyperacusis?
Can early diagnosis reduce the risk of hyperacusis getting worse?
What is the natural history (onset and changes over time) of hyperacusis?
Are hearing aids (for managing hearing loss) an effective treatment for hyperacusis?
Is there an association between hearing loss and hyperacusis?
Is peer support (e.g. support groups, mentors, families) effective for management of hyperacusis?
Does avoidance of sound improve hyperacusis or make it worse?
Is stress management effective for hyperacusis?
Is hyperacusis a symptom, a condition or a disability?
What is the level of public/employer awareness of hyperacusis?
What should be the maximum volume (e.g. in movies, concerts, headphones) to accommodate people with hyperacusis?
Can noise exposure cause hyperacusis (or make it worse)?
What preventive measures (e.g. educational information, warning labels) reduce the risk of hyperacusis developing or getting worse?
Are there certain personality traits in those prone to hyperacusis?
Which management approach (e.g. assessment and treatment) is most effective for people with hyperacusis and dementia?
What are the risk factors for developing hyperacusis or making it worse?
Is there a link between hyperacusis and non-ear-related conditions (e.g. Fibromyalgia, multiple sclerosis, diabetes, chronic fatigue, pernicious anaemia)?
Which health professionals should work together to provide the best care for children with hyperacusis?
What is the essential knowledge/training required for health professionals to appropriately refer or effectively manage hyperacusis?
Which psychological therapy (e.g. counselling, Cognitive Behavioural Therapy, mindfulness) is most effective for hyperacusis?
Is hyperacusis associated with middle ear pathology?
What is the relationship between hyperacusis and autism?
What is the relationship between mental health and hyperacusis?

For peer review only

Is hyperacusis linked to other sensitivities/conditions?
Is there an association between hyperacusis and other ear-related conditions (e.g. superior canal dehiscence syndrome, Meniere's, Waardenburg syndrome, vertigo, vestibular migraines)?
Is hyperacusis linked with trauma (e.g. brain or neck trauma, a traumatic event)?
Does stress cause or make hyperacusis worse?
Why does hyperacusis fluctuate?
What is the role of fear in hyperacusis?
Which drugs cause hyperacusis?
Is there a genetic element to hyperacusis?
What is the best work/study/healthcare environment to support people with hyperacusis?
Are any complementary (relaxation, yoga, meditation, hypnotherapy) or alternative therapies (laser therapy, vibration therapy) effective for hyperacusis?
Is hyperacusis related to physical changes in the ear or brain?
Is hyperacusis due to physical or psychological issues or is it a combination of both?
What is the best information and guidance for parents dealing with hyperacusis in their children?
What area(s) of the brain and patterns of activity is/are associated with hyperacusis?
Can otitis media with effusion cause hyperacusis?
Can infections (e.g. measles, yeast, viral) cause hyperacusis?
Can ear care (suctioning, syringing, swabbing) cause hyperacusis?
Do dental problems cause hyperacusis?
What is the best way to differentiate hyperacusis from other hearing conditions (e.g. recruitment, misophonia, Meniere's, tinnitus)?
Can hyperacusis be caused or made worse (e.g. by hormonal changes) in pregnancy?
Do musculoskeletal problems cause hyperacusis?
What are the 'red flags' for serious underlying conditions in hyperacusis?
Which criteria should be met to diagnose hyperacusis in adults/children?
Do any drugs prevent hyperacusis?
Which self-help interventions are effective for hyperacusis?
What management approach for hyperacusis is most effective for adults/children with autism?

For peer review only

Just about finished…a little bit more about you…

It is important we find out about a little about you, to make sure that we have a wide range of people with different experiences complete the survey.

What is your age or the age of your child with hyperacusis?

- ☐ under 10
- ☐ 10 - 20
- ☐ 21 - 30
- ☐ 31 - 40
- ☐ 41 - 50
- ☐ 51 - 60
- ☐ 61 - 70
- ☐ 71 - 80
- ☐ 80+

How would you describe your gender?

- ☐ Male
- ☐ Female
- ☐ I would describe it in another way

Healthcare professional only: What is your main profession?

For example, GP, ENT, Audiologist, clinical psychologist.

- ☐ Audiologist
- ☐ Audiovestibular Physician
- ☐ Chiroptactor
- ☐ Clinical Psychologist
- ☐ Clinical scientist
- ☐ ENT
- ☐ Hearing therapist
- ☐ Hynotherapist
- ☐ Paediatric audiologist
- ☐ Paediatrician
- ☐ Psychiatrist
- ☐ Nutritionist
- ☐ Other____

For peer review only

Thank you for completing this survey

Would you like to help us in the next stage of this study?

The final stage of project will be a face-to-face consensus meeting with 30 people to discuss the results from this survey and agree the Top 10 research questions. We are delighted to announce that this meeting will be held in the picturesque surroundings of the University of Nottingham (UK) on Monday 23rd July (10.00 - 17.00). **We need people with lived experience of hyperacusis, parents, carers, teachers, members of the public and healthcare professionals with experience of hyperacusis to participate in this meeting.** If you live in the UK and would like to participate, please contact us here to register for the meeting: JLAhyperacusis@nottingham.ac.uk. Places are available on a first come, first served basis. Travel for the meeting will be reimbursed and accommodation will be provided.

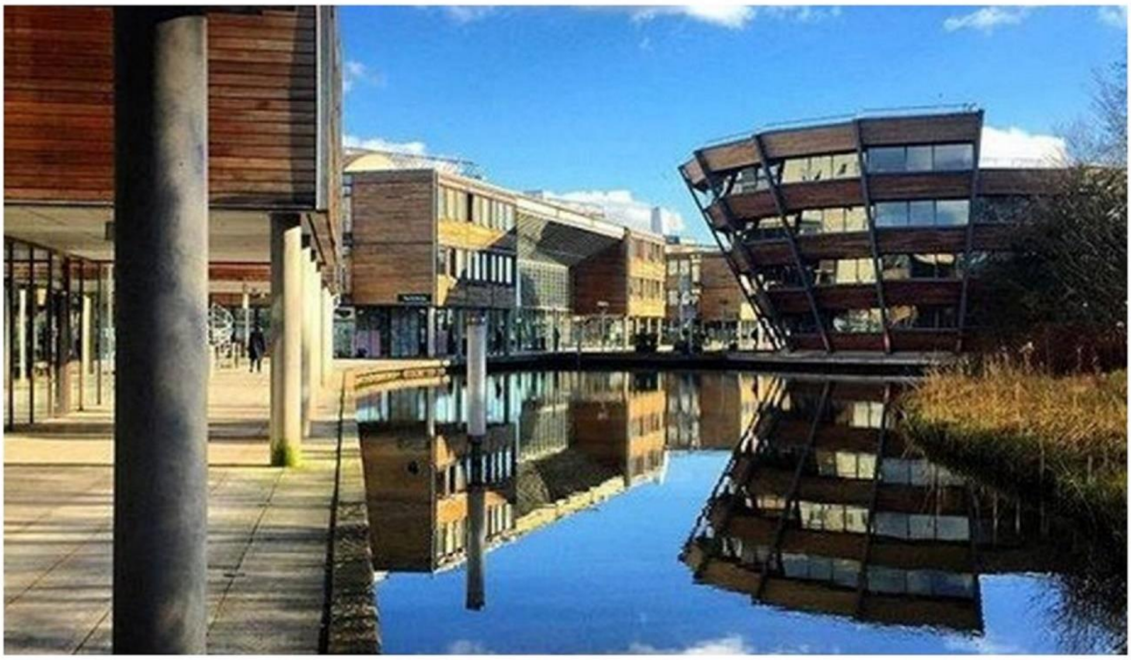

Once again thank you for your support on this project.

Please follow us on twitter for updates @HyperacusisJLA.

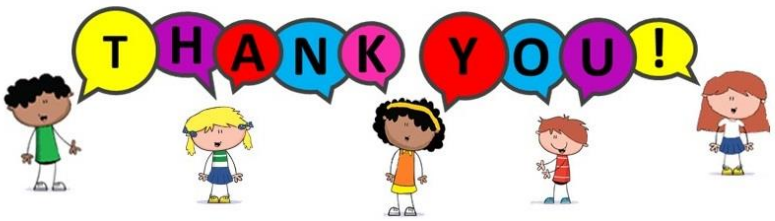

Privacy Notice

Here at the Hyperacusis James Lind Alliance Priority Setting Partnership (PSP) we take your privacy seriously. If you choose to send us your name and preferred contact details, we will only use this information for the purposes of contacting you to invite you to participate in future activities related to this PSP, which you might be interested in. There are no consequences to you if you do not want to provide your name and contact details.

The lawful basis for holding this data is that there is a legitimate interest for you to be contacted about future PSP activities. This helps the PSP ensure it listens to a variety of perspectives. The only people who have access to your name and contact details is the Administrator of this PSP and the research Lead. Your name and contact details are not linked to the survey in any way, and are not shared with anyone else. Your information is not subjected to any automatic processing or decision making.

Your name and contact details will be deleted at latest when the activities of this PSP close in August 2018. You have the right to see what information we hold about you, to correct it, and to ask us to delete it, at any time.

If you would like any further information or have any questions, please contact the PSP Administrator, Kathryn Fackrell on JLAhyperacusis@nottingham.ac.uk or look at our website: http://www.hearing.nihr.ac.uk/research/hyperacusis-PSP

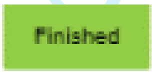

Supplemental appendix 4: Categories and sub-categories with example questions

**Categories**<br>*Sub-categories*	**Example question**
**Causes/Risk factors**	
Genetics	Are there genetic or epigenetic factor that impact the likelihood of getting hyperacusis?
Pregnancy	Can hyperacusis be caused by hormonal changes in pregnancy?
Physical or psychological	Is it triggered by worry about hearing a loud noise or can a loud noise in itself trigger the condition?
Nerve damage	Can muscular damage or wear and tear to jaw joints or neck cause hyperacusis?
Treatment	Does the insertion of cotton swabs deep inside the ear canal cause hyperacusis?
Infections	Does glue ear cause hyperacusis?
Diet/Exercise	Does diet affect hyperacusis?
Behaviour	Can hyperacusis be learned?
Exposure	Can exposure to loud sounds worsen or cause hyperacusis?
**Exacerbation**	Is there proof that overprotecting exacerbates hyperacusis?
**Classification**	Should hyperacusis be categorised differently based on its underlying causes rather than assuming they're all related?
**Prevalence**	What are the percentages of co morbidity across these 3 conditions (hyperacusis, hearing loss and tinnitus)?
**Natural history**	What is the natural progression/prognosis?
**Mechanisms**	What role, if any, does the cochlear feedback mechanism have in hyperacusis?
**Links with other conditions**	
Attention Deficit & Hyperactivity disorder	Is hyperacusis associated with other medical conditions/disabilities such as ADHD?
Autism	Is there a link between Autism and hyperacusis?
Dementia	Is hyperacusis or a type a hyperacusis a common experience in dementias?
Hearing	What is the relationship between hyperacusis and hearing loss?
Non-ear-related	Is there links between hyperacusis and chronic fatigue syndrome?

For peer review only

Other sensitivities	Do people with hyperacusis have other CNS symptoms ...e.g. sensitivity to bright light?
Personality	Does personality affect patients with hyperacusis?
Mental health	What role does depression and anxiety, and other mental health issues, play in how it affects people?
Trauma	Is it connected to head injury?
**Assessment**	What audiological test battery can be used by audiologists to detect hyperacusis and help monitor progress or regression in their patient's follow up consultation sessions?
**Management**	
Amplification	Can there be a hearing aid that has some kind of built-in equalizer so that certain frequencies can be lowered/adjusted before they reach ears?
Associated conditions	How can management of hyperacusis be reasonably adjusted for adults with intellectual disabilities?
Complementary/ alternative therapies	Does yoga improve hyperacusis?
Digital intervention	How useful /effective is online treatment for hyperacusis?
Effective treatment (general)	What is the efficacy of the current treatment strategies?
Medication	Can Selective Serotonin Reuptake Inhibitors help adults with hyperacusis?
Self-management	What can I do in my own time while I wait for the professional treatment to start, to speed up recovery?
Sound therapy	Are sound generators effective in the management of hyperacusis in children?
Surgical	How does a stapedectomy cause hyperacusis?
Tinnitus Retraining Therapy	Does TRT based treatment help hyperacusis in the long term?
Psychological therapy	Does cognitive behavioural therapy help manage underlying anxiety to further reduce hyperacusis in patients?
Dental	In what ways can we alter dental treatment to make the experience less painful for those suffering from hyperacusis?
**Prevention**	Can hyperacusis be prevented by warning labels in concerts, album covers, music apps, headphone packaging, etc.?
**Awareness**	How to roll out better education of the condition once diagnosed for families?
**Schools**	Should children with hyperacusis receive any additional support or accommodations at school?
**Service provision & evaluation**	Should there be a joint psychology/ audiology approach to managing hyperacusis?-
**Training**	Is specialty training available to otolaryngologists or audiologists regarding hyperacusis?

For peer review only

**Support**	Do those who experience Hyperacusis benefit from attending support groups?

For peer review only
